# Software and Computational Tools for LC-MS-Based Epilipidomics: Challenges and Solutions

**DOI:** 10.1021/acs.analchem.2c04406

**Published:** 2023-01-10

**Authors:** Tito Damiani, Stefano Bonciarelli, Gerhard G. Thallinger, Nikolai Koehler, Christoph A. Krettler, Arif K. Salihoğlu, Ansgar Korf, Josch K. Pauling, Tomáš Pluskal, Zhixu Ni, Laura Goracci

**Affiliations:** 1 Institute of Organic Chemistry and Biochemistry of the Czech Academy of Sciences, Flemingovo nám. 2, 160 00 Praha 6, Czech Republic; 2 Department of Chemistry, Biology and Biotechnology, University of Perugia, Via Elce di Sotto 8, 06123 Perugia, Italy; 3 LipiTUM, Chair of Experimental Bioinformatics, Technical University of Munich, Maximus-von-Imhof Forum 3, 85354 Freising, Germany; 4 Enveda Biosciences, Boulder, Colorado 80301, United States; 5 Department of Physiology, Faculty of Medicine and Institute of Health Sciences, Karadeniz Technical University, 61080 Trabzon, Turkey; 6 Bruker Daltonics GmbH & Co. KG, Fahrenheitstraße 4, 28359 Bremen, Germany; 9 Center of Membrane Biochemistry and Lipid Research, University Hospital and Faculty of Perugia, Via Elce di Sotto 8, 06123 Perugia, Italy; 10 Institute of Biomedical Informatics, Graz University of Technology, 8010 Graz, Austria

Lipids play a crucial role in cellular structure and functions, including cell signaling, membrane plasticity, and trafficking. Alterations of the lipid composition in cells, tissues, or organelles have been associated with a large number of diseases, including inflammation, cancer, and degenerative or metabolic disorders.^1−5^ Within biological systems, lipids can undergo a range of enzymatic and nonenzymatic reactions (e.g., oxidation, nitration, sulfation, and halogenation) that introduce structural modifications and/or new functional groups to the native molecule.^6^ The resulting modified lipid species, generally referred to as “epilipids”, are known to be heavily involved in the regulation of physiological and pathological conditions.^7−16^

Several analytical approaches have been applied to lipids analysis and have been extensively reviewed elsewhere.^17−19^ Among them, high-resolution mass spectrometry (MS) currently represents the most popular technique due to the unprecedented sensitivity, mass accuracy, and resolving power offered by modern MS instruments.^20^ In addition, separation techniques such as liquid chromatography (LC) can be conveniently coupled to the MS analyzers to attain a prior sample separation that reduces ion suppression phenomena and increases signal-to-noise ratio for low-abundance analytes.^21,22^ Moreover, the recent development of MS devices with ion mobility spectrometry (IMS) can support further characterization of the measured lipid species and increase the identification confidence in untargeted studies.^22^ Despite these technological advances, only a few epilipid classes (e.g., eicosanoids and docosanoids)^8,23^ have been extensively studied over the past decade, and our global understanding of the “epilipidome” (bio)chemistry is still very limited.^15,24^ The main obstacle to a comprehensive epilipidome analysis and annotation arguably lies in its intrinsic chemical complexity, which causes a series of both analytical- and computational-related challenges:

(1) *Low abundance*. Epilipids generally occur with an inherently low abundance and transient nature within biological systems (e.g., the absolute amounts/concentrations of lipid oxidation products *in vivo* are estimated in the order of 0.03–3.0 mol % of the total nonoxidized lipidome).^25^ As a consequence, despite the exceptional sensitivity reached by modern MS detectors, the signal produced by modified lipids is often close to the instrumental limit of detection.^16^ This represents a major obstacle to the use of shotgun and/or MS-imaging methods in epilipidomics studies as they suffer from low overall sensitivity due to scarce ionization efficiency of low-abundance metabolites. Hyphenated techniques (e.g., LC-MS) not only provide an additional separation dimension but also can increase the overall dynamic range by reducing ion suppression effects. For these reasons, LC-MS currently represents the technique of choice for the study of epilipids.

(2) *High structural diversity*. The nature and likelihood of lipid modifications are largely determined by the molecule’s chemical structure. In fact, most of the reactions occur at energetically favorable sites of the molecule.^26^ For example, double bonds are deemed the most susceptible sites to electrophilic addition in polyunsaturated fatty acids (PUFAs), while allylic and bis-allylic positions are prone to radical attack. In addition, chemical modifications may also involve the lipid headgroup, vinyl ether moieties, and ester functional groups.^27−29^ Considering the lipids’ remarkable structural diversity, the large number of potential modification sites and the range of potential reactions (e.g., oxidation, nitration, sulfation, etc.), an enormous number of chemically distinct derivatives is possible.^26^ In this regard, Ni et al. estimated the epilipidome size by knowledge-based and systematic enumeration, only considering a few main oxidative modifications (Figure 1).^16^ Indeed, if an algorithm tries to enumerate epilipids with complete permutations with repetition of all modifications at all possible allylic and bis-allylic positions (approach A in Figure 1), a vast search space with over 10^12^ species results. This space includes a large number of unnatural structures. In contrast, if knowledge-based algorithms are used to estimate only actually possible modifications reported for enzymatic and free radical based mechanisms of fatty acids (FAs) (approach B in Figure 1), a more biologically relevant estimation of possible product space with less than 10^5^ enumerated structures will result. Nevertheless, the scheme in Figure 1 well represents the high structural diversity within the epilipidome and makes clear that the generation of a comprehensive database of epilipids to be used for identification purposes (a common approach for native species) is hardly applicable.

**Figure 1:**
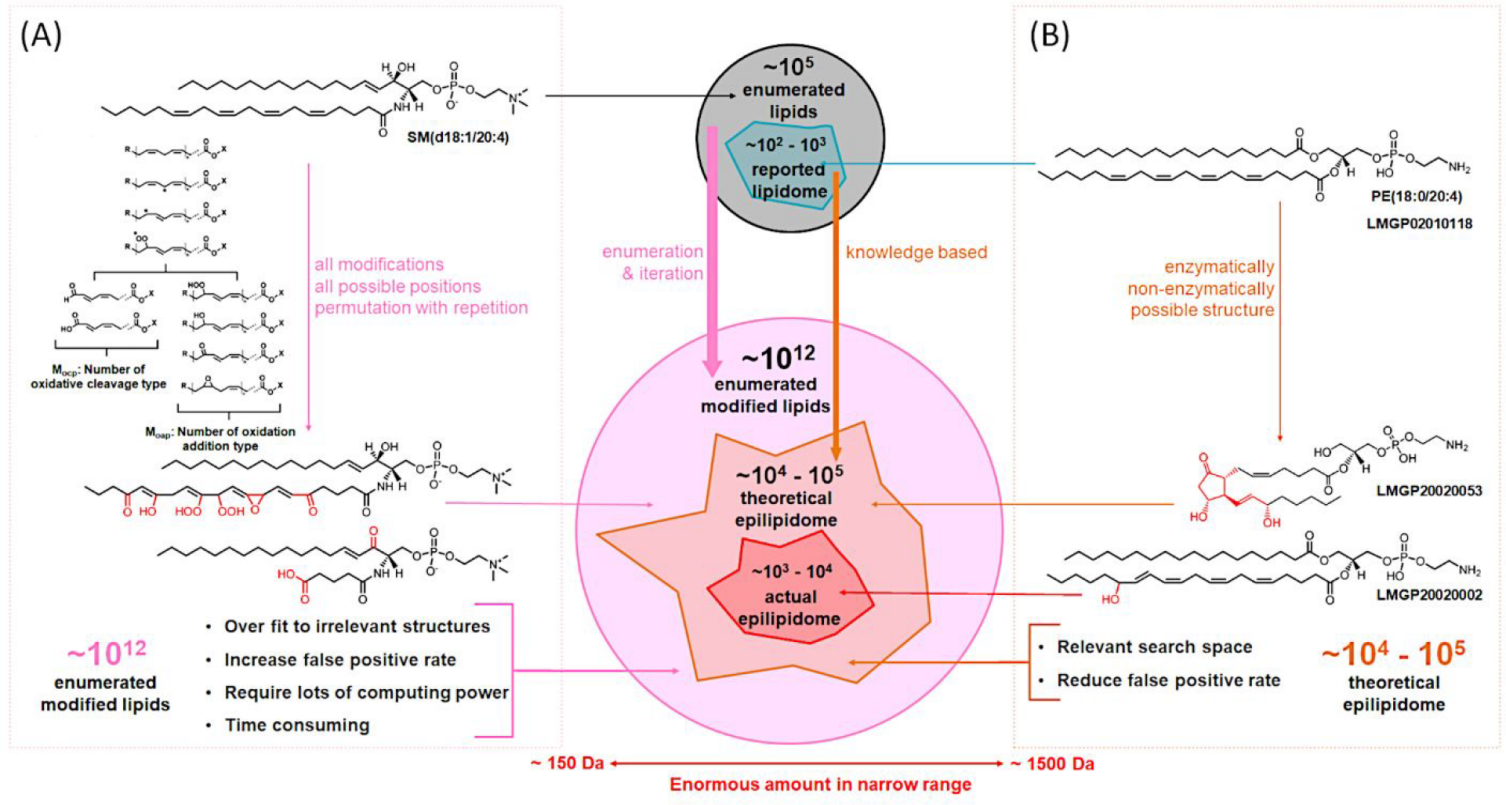
Epilipidome chemical space within the molecular weight range of 150–1500 Da. (A) Combinatorial enumeration of all permutations with repetition of all modifications at all possible allylic and bis-allylic positions. In this example, a lipid species which is not reported in common databases such as SM(d18:1/20:4) can be constructed and selected as a precursor for oxidation, aiming at enumerating all possible modifications at all possible positions. (B) Knowledge-based enumeration of only actually possible modifications of fatty acid residues on certain sites reported for enzymatic and free-radical based mechanisms. As an example, the widely reported oxylipin products from arachidonic acid can be used to predict oxidation addition products from commonly reported phospholipids with arachidonic acid residue such as PE(18:0/20:4) and generate products with oxylipin residue such as PE(18:0/15-HETE) and PE(0:0/PGE2) which are confirmed by literature.^30,31^ The figure is adapted from Ni, Z.; Goracci, L.; Cruciani, G.; Fedorova, M. Computational Solutions in Redox Lipidomics - Current Strategies and Future Perspectives. *Free Radic Biol Med***2019**, 144, 110–123^16^ under the terms of the Creative Commons Attribution 4.0 International License. To view a copy of this license, visit http://creativecommons.org/licenses/by/4.0/. The original script for epilipidome estimation is available on GitHub (https://github.com/SysMedOs/LipidomeEstimation).

(3) *Large number of isomers/isobars*. A direct consequence of the vast chemical space covered by the epilipidome is the significant number of isobaric (same nominal mass) and isomeric (same exact mass) species. As an example, querying the exact mass 649.40 with a tolerance of ±0.05 Da in the LIPID MAPS Structure Database (LMSD)^32^ returns three distinct oxidized phosphocholines characterized by different elemental composition (PKHdiA-PC [LMGP20010009], PHOOA-PC [LMGP20010016], and PON-PC [LMGP20010008]; Figure 2A). Correspondingly, searching a specific elemental formula within the same database returns multiple isomers with the same exact mass but different chemical structures (P-IsoPGE2-PC [LMGP20010043], P-LGE2-PC [LMGP20010049], and P-LGD2-PC [LMGP20010050]; Figure 2B). High-resolution MS systems can nowadays provide high mass accuracy and sufficient resolving power to distinguish isobaric species; however, the problem persists for structural isomers, which exhibit identical exact mass and isotopic pattern. In this case, a confident annotation can be achieved only by inspecting the collected fragmentation spectra.

**Figure 2:**
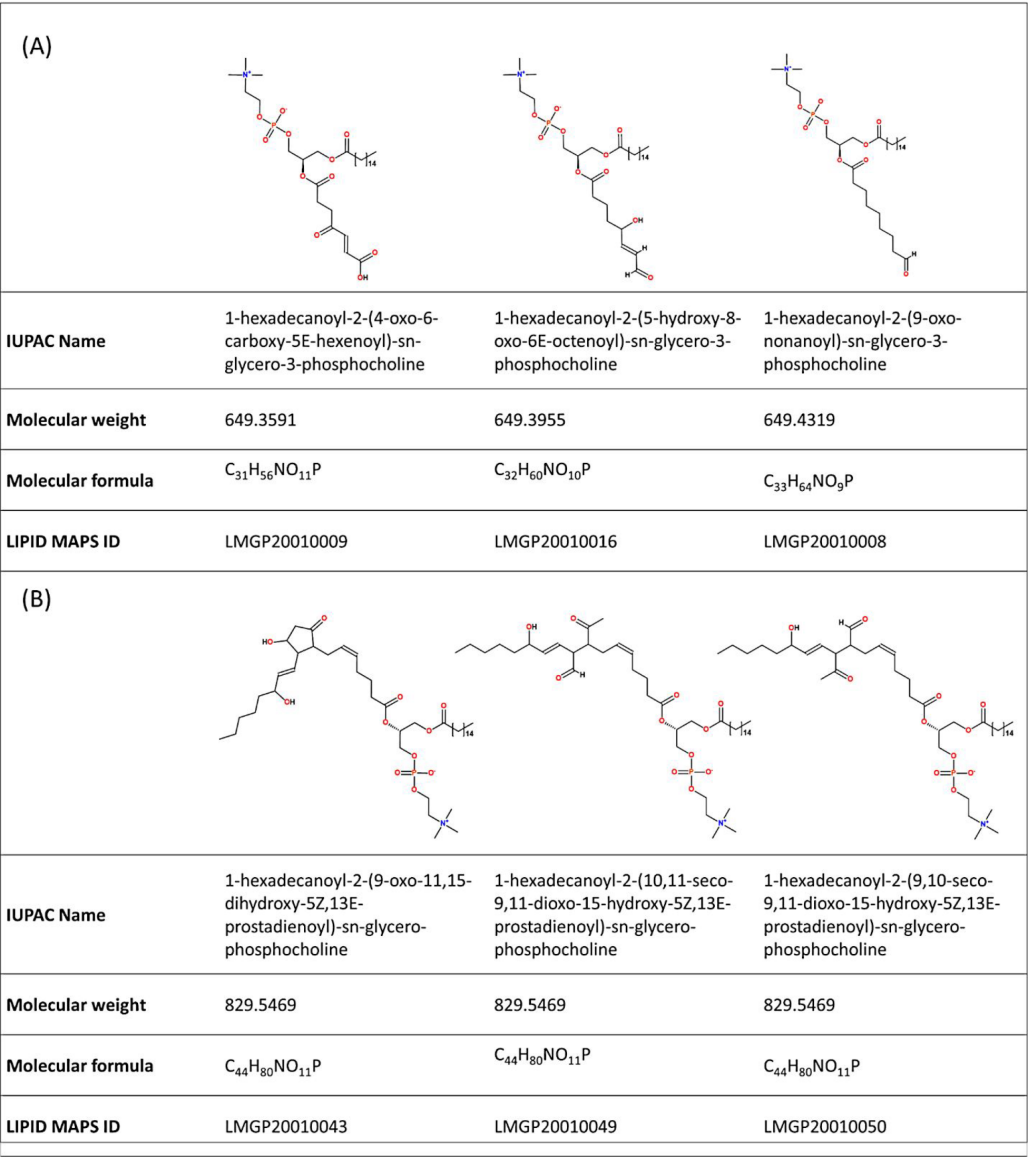
Examples of isobaric and isomeric lipid species in LIPID MAPS. (A) Three isobaric oxidized phosphatidylcholines obtained by a search for a mass of 649.40 ± 0.05 Da. (B) Three isomeric oxidized phosphatidylcholines obtained by a search for C_44_H_80_NO_11_P (accessed September 2022).

(4) *Different fragmentation compared to native lipids*. Fragmentation mass spectra (also referred to as MS/MS spectra) can be used as “molecular fingerprints” to assign a tentative annotation to the unknown detected metabolites.^33^ Reference libraries of MS/MS spectra can be used to automatize the metabolite annotation using a spectral matching approach (i.e., experimental MS/MS data are compared with the reference spectra and the annotation assigned based on the best match). Although this currently represents the most effective approach for metabolite annotation,^34,35^ two main obstacles severely limit its application to epilipids. First, comprehensive collections of commercially available analytical standards are currently lacking for these species. This prevents the acquisition of reference MS/MS spectra from the pure compounds and their integration in existing libraries. Second, the fragmentation patterns of epilipids may significantly differ from those of parent molecules. In fact, even “small” modifications in the lipid chemical structure can produce substantial differences at the MS/MS fragmentation level. For instance, both 5-hydroxy-PGI1 and 6-keto-PGF1α originate from 5Z,8Z,11Z,14Z-eicosatetraenoic acid (arachidonic acid) and even share the same elemental composition (i.e., C_20_H_34_O_6_); nevertheless, they produce significantly different fragmentation spectra.^36^ As a consequence, MS/MS libraries of native lipids cannot be effectively exploited for the annotation of modified species as it would produce unreliable results.

(5) *Nomenclature*. Lipid nomenclature has historically represented an arduous task due to the lipidome’s structural diversity and complexity.^37−40^ A systematic shorthand notation for MS-based lipid structure annotation has evolved in the past two decades to allow their unequivocal reporting in a clear and succinct way.^41,42^ However, despite the recent efforts of LIPID MAPS,^37,38^ a unified nomenclature scheme for modified lipid species throughout all the lipid categories is still lacking. As a consequence, improper annotation and over-reporting is commonly found among research papers.^43^ As an example, 1-palmitoyl-2-(9-oxo-nonanoyl)-*sn*-glycero-3-phosphocholine (LMGP20010008) has been reported with at least six different, but similar names in recent publications.^44−49^ Although these names may be very similar and/or differ only in one character, a simple string matching would fail when parsing lipid names (e.g., querying databases). This reduces the usability of reference data and complicates the unified computational treatment of lipid names.^40,50^ Common abbreviation systems have been adopted by the community only for a few oxylipin species (e.g., 15-HETE, 12(R)-HpETE, and PGE2).^51−53^ However, these abbreviations carry almost no structure-related information and require the researcher to possess prior knowledge for their correct interpretation.

Although the above-mentioned challenges are currently hampering the study of the epilipidome through MS-based methods, they can be addressed (or at least mitigated) by software-assisted data analysis pipelines. Dedicated tools have started to be available; yet, the information on the existing solutions is rather unsystematic, hindering their large-scale implementation by the community. In this context, the present review focuses on the computational tools and approaches currently available for the analysis of LC-MS epilipidomics data. In the following sections, a general epilipidomics LC-MS workflow is described with a focus on the data analysis steps as well as the challenges that computational tools are expected to overcome (analytical approaches for epilipids analysis were recently reviewed elsewhere^54,55^). We placed our emphasis on GUI-based software as they provide more usable solutions for researchers lacking programming skills.^56^ Finally, emerging computational tools that we believe can support the analysis of LC-MS epilipidomics data are described.

## LC-MS-Based Epilipidomics Data Analysis

**Figure 3:**
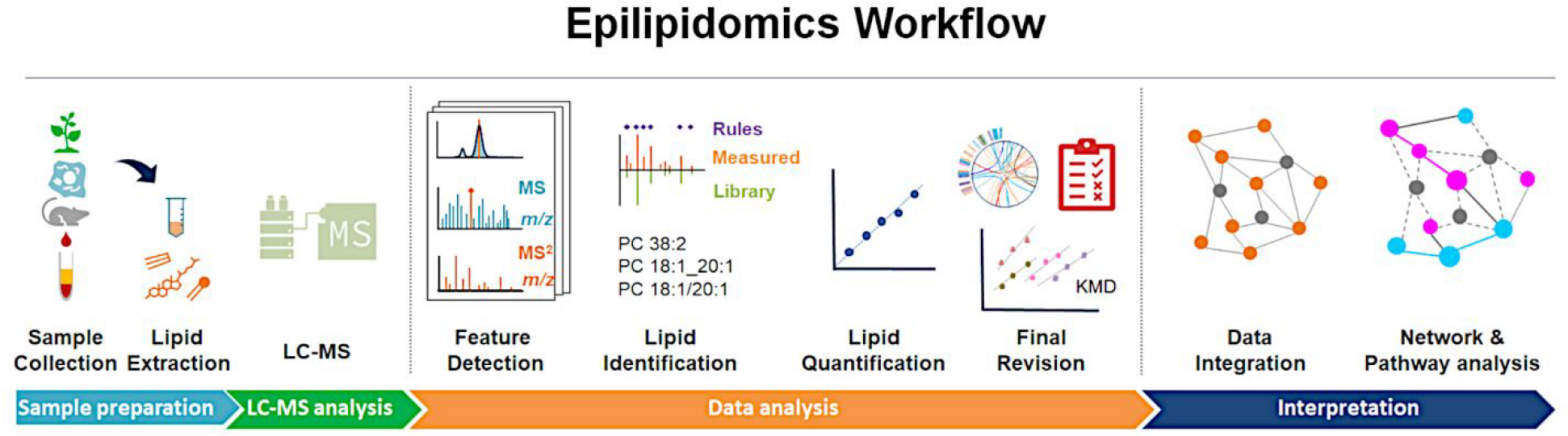
Scheme of the (epi)lipidomics workflow described in four stages: sample preparation, LC-MS analysis, data analysis, and interpretation.

In this section a general workflow of an LC-MS-based (epi)lipidomics study including all data processing steps is described (Figure 3). We divided the data analysis stage into four main steps: *feature detection*, *identification*, *quantification*, and *results investigation*. A detailed description of each of these steps is provided below. Emphasis is placed on how the above-discussed challenges translate into computational problems that prevent the ready implementation of lipidomics-optimized pipelines into epilipidomics workflows.

### Feature Detection

In LC-MS-based *omics* experiments, the term *feature* typically refers to a two-dimensional signal (i.e., peak) that represents a chemical compound. The first step in untargeted LC-MS data processing is normally the “feature detection” (a.k.a. “peak picking”), where boundaries (*m*/*z* and retention time values) and intensities are determined for all the features detected in the analyzed samples.^57^ The final goal is to turn the complex LC-MS raw data into a list of detected features ready for further downstream data analysis.^57,58^*Feature detection* normally involves several consecutive substeps, including extracted ion chromatograms (EICs) construction and deconvolution, feature filtering (e.g., deisotoping), alignment, etc. We identified two of these substeps as potential pitfalls when dealing with epilipidomics data.

First, during the EIC building step, the user is normally asked to set a noise level threshold which determines the minimum signal intensity (i.e., area or height) for an LC peak to be retained as a *feature*. Intuitively, this threshold has to be chosen with particular care in epilipidomics applications, due to the natural low abundance of modified lipid species. In fact, an overly high noise cutoff will lead to the erroneous discarding of relevant signals. On the other hand, an overly low noise threshold unavoidably produces an exponentially larger number of background signals to be retained in the final data table, which extends the computation time/cost and might complicate downstream processing steps. This problem can be mitigated by fine-tuning the *feature detection* algorithm parameters and by setting additional constraints (e.g., minimum number of data points, signal/noise ratio, etc.) for an LC signal to be considered an actual *feature*.^59−62^

Second, untargeted LC-MS analyses typically rely on generic chromatographic column chemistries and gradients. Under these conditions, positional isomers often exhibit similar chromatographic behavior, producing coeluting and/or shoulder peaks. The EICs deconvolution step aims at correctly splitting neighboring LC peaks that do not present a full baseline separation. Although robust algorithms have been developed for this purpose,^60,62−64^ manual tuning and visual inspection of the results is usually needed to ensure results fidelity. However, this requires the user to have a good understanding of the (often several) algorithm parameters to be set, since even little changes in these settings have been shown to have a dramatic impact on the results.^65,66^

### Lipid Identification

Lipid annotation is unanimously recognized as the primary bottleneck in (epi)lipidomics studies.^16^ MS-based identification of lipids can be carried out at six levels of structural information as described by Liebisch et al.,^37^ with the highest achievable level being the “fatty acyl/alkyl/sphingoid base structure”. As for the annotation of small molecules in general, assignment of the elemental formula to the (unknown) *m*/*z* signals of interest is the first committed step toward its structural identification.^67^ As discussed above, the epilipidome covers a remarkably vast chemical space over a relatively narrow molecular weight range, which inevitably leads to the ample occurrence of isobaric species. Therefore, the sole exact mass match is usually not sufficient for an unequivocal elemental composition assignment as many candidates would fall within the mass range search. In this respect, the isotope pattern information represents an irreplaceable means to rule out incorrect candidate formulas and raise the annotation confidence.^68^ The next step is the tentative annotation of the chemical structure of (epi)lipid based on the collected MS/MS data. The fragmentation spectra are either compared to reference spectral libraries or annotated using a rule-based approach.^16,69^ However, epilipids are currently underrepresented in reference MS/MS spectral libraries (see Databases Containing Epilipids) due to the scarcity of commercially available standards. To circumvent the need for comprehensive reference standard sets, libraries of MS/MS spectra can be simulated *in silico*, using well-characterized fragmentation rules (i.e., class-specific fragment ions and neutral losses) and subsequently matched against the experimental spectra.^70,71^

Regardless of the annotation strategy, multiple structure candidates (especially structural isomers) are normally returned.^72^ In this regard, additional separation dimensions prior to the MS acquisition can provide valuable information to raise the annotation confidence. For instance, a chromatographic separation can avoid coeluting isomers being cofragmented and prevent the generation of chimeric MS/MS spectra. Annotation constraints based on RT and/or IMS-derived collisional cross section (CCS) information can be included in the identification strategy to filter out incorrect candidate structures and reduce the false discovery rate.^72,73^

### Lipid Quantification

Following the identification step, the analyst is often interested in the absolute or relative quantification of the annotated metabolites across the analyzed samples.^74^ Targeted applications rely on calibration curves and/or the addition of internal standards for the accurate quantification of the analytes of interest,^75^ although this is clearly limited to those molecular species for which analytical standards are available. A semiquantitative approach can be pursued by using a single internal standard per lipid class.^76^ In the case of modified lipids, using analytical standards from the same subclass species (ideally the same type of modification) could represent a viable alternative. In practice, untargeted studies do not normally require absolute quantification levels and the measurement of the analyte abundance is simply based on the chromatographic peak area or height.^77^ In this regard, it must be taken into account that a given analyte can generate multiple ion species (e.g., [M + H]^+^ adduct, [M + Na]^+^ adduct, in-source fragments, etc.) that will be recognized as distinct features due to the different parent *m*/*z*. As a result, the overall signal will be distributed over multiple entries in the final feature list. Therefore, relying on a single adduct for the feature quantification could introduce biases in the downstream analysis. The number and type of generated adducts do not depend only on the chemical structure and characteristics of the individual molecule, but also on experimental variables difficult to control (e.g., solvent purity). Moreover, certain structural modifications can change the ionization behavior and efficiency with respect to the native species (e.g., a different adduct distribution can be favored).^44,78^ For instance, the majority of oxidized phosphatidylcholines (oxPCs, e.g., LMGP20010003 and LMGP20010005) are generally detected as formate or acetate adducts, while truncated oxPCs with terminal carboxylic moieties (e.g, LMGP20010006 and LMGP20010007) tend to form [M–H]^−^ adducts.^44^ Algorithms for the automatic grouping of multiple ion species produced by the same chemical entity can address this problem. In the specific case of epilipids, tools able to “dynamically” pick the correct adduct species for quantification would be highly desirable.

### Results Investigation

One of the ultimate goals of untargeted LC-MS data analysis pipelines is to enable an informative visualization of the annotation and quantification results. Various data displaying strategies have been proposed over the years to inspect identification results, spot misannotations, etc. In this section, we report two data visualization tools developed in different research fields and later adapted to (epi)lipidomics applications: Kendrick mass defect and Circos plots.

**Figure 4:**
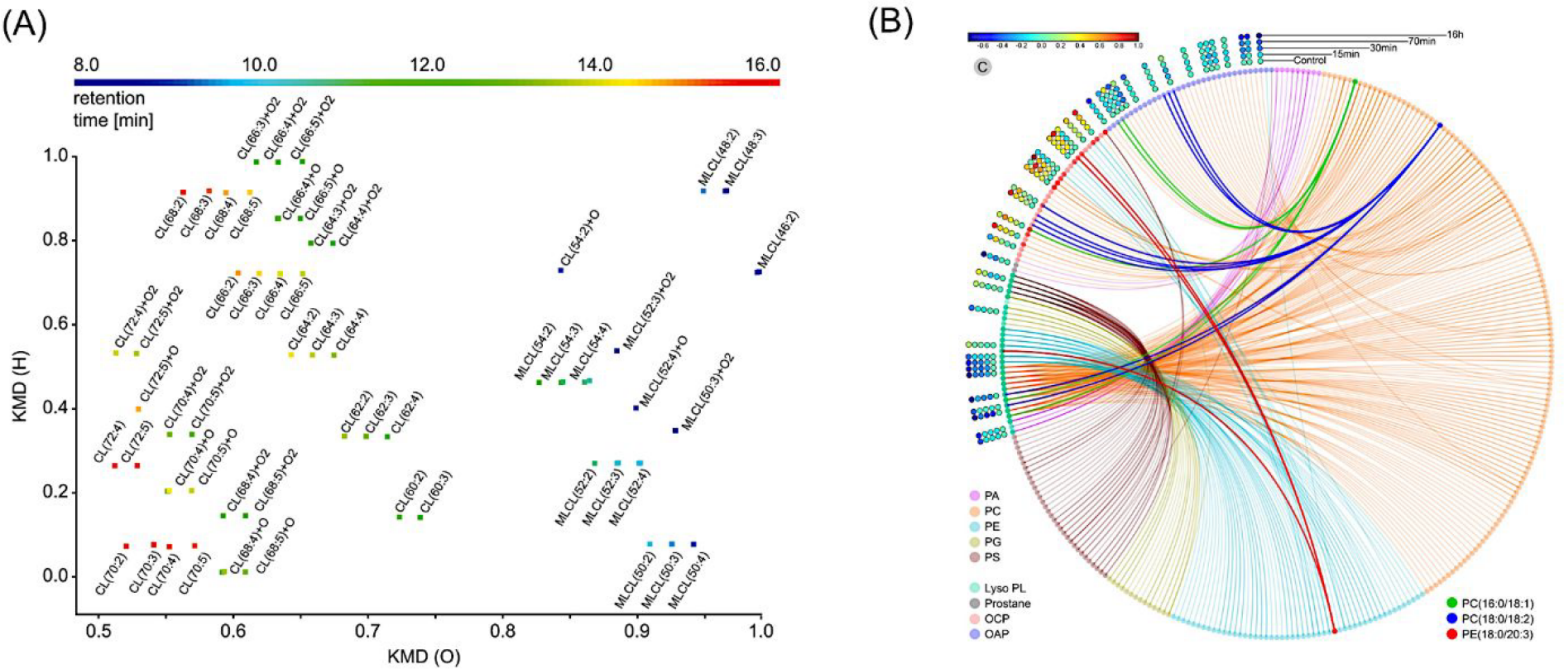
Visualization of epilipid identification results. (A) KMD plot for cardiolipins (CL), monolysocardiolipins (MLCL), and their corresponding oxidation products. The difference in saturation and the degree of oxidation are displayed on the *y*-axis (i.e., KMD(H)) and *x*-axis (i.e., KMD(O)), respectively. The RT dimension is represented as a color-coded scale. The figure is adapted from Helmer et al.,^83^ under the terms of the Creative Commons Attribution 4.0 International License. To view a copy of this license, visit http://creativecommons.org/licenses/by/4.0/. (B) Circos diagram illustrating the relationship between the identified/quantified oxidized and parent phospholipid species. (PA: Glycerophosphates; PC: Glycerophosphocholines; PE: Glycerophosphoethanolamines; PG: Glycerophosphoglycerols; PS: Glycerophosphoserines; Lyso PL: Monoacylglycerophospholipids; OAP: Oxygen addition products; OCP: Oxidation cleavage products). The figure is adapted from Ni, Z.; Angelidou, G.; Hoffmann, R.; Fedorova, M. LPPtiger Software for Lipidome-Specific Prediction and Identification of Oxidized Phospholipids from LC-MS Data sets. *Sci Rep***2017**, 7 (1), 15138^44^ under the terms of the Creative Commons Attribution 4.0 International License. To view a copy of this license, visit http://creativecommons.org/licenses/by/4.0/.

The Kendrick mass defect (KMD) plot is a graphical data representation designed to assist the identification of compounds that include repeating units in their chemical structures.^79,80^ The KMD plot enables the representation of the whole LC-MS data set in a single chart, where compounds containing repeated structural features form easily recognizable homologous series. Briefly, a repeating unit of interest (e.g., CH_2_) is chosen, and the corresponding Kendrick mass factor is computed as the ratio between its nominal and exact mass. This factor is then used to create the Kendrick mass scale and calculate the Kendrick mass defect. The KMD plot is then generated by plotting the KMD of the selected structural feature against the *m*/*z* or the Kendrick mass. More details about “Kendrick analysis” in general can be found in Fouquet, 2019.^81^ Although originally developed for petroleum analysis,^79,81^ KMD(CH_2_) and/or KMD(H) can be used to highlight differences in the acyl chain length and saturation of homologous lipid species.^82−85^ Korf et al. created a KMD plot by plotting two KMDs against each other (i.e., CH_2_ on the *x*-axis and H on the *y*-axis) and used it to identify more than 100 lipid species in*Chlamydomonas reinhardtii*.^82^ In the case of epilipids, the specific modification can be used to identify modification products of a lipid class in complex samples. For instance, Helmer et al. plotted the KMD(H) against the KMD(O) to display cardiolipin oxidation products analyzed via LC-MS (Figure 4A).^83^ The chromatographic information (i.e., RT) can be also integrated in the KMD plot in the form of a color-coded scale to enable further visualization possibilities.^83^ The resulting three-dimensional KMD plot can be particularly useful to confirm identification results and/or recognize false annotations. In fact, clear class-specific trends are often observable depending on the applied chromatography (i.e., hydrophilic interaction or reverse-phase chromatography).^82,83^

The Circos plot is a visualization approach for displaying complex data using highly customizable, information-rich circular layouts. More details can be found in Krzywinski et al.^86^ Although originally created for genomic data visualization,^86^ Circos diagrams can be adapted to other data types and have recently found applications in many different fields, including epilipidomics. For example, Jha et al. employed the Circos plot to visualize 96 hepatic lipid measured in 385 mice livers and their fold change between control and treated groups.^87^ Multilayered annotations were also added to highlight the significant intergroup correlations, including the strength and approximate chromosomal position of the identified quantitative trait loci. Besides generic packages designed to create Circos plot with any type of data,^88,89^ Ni et al. developed the Python package LipidCircos for the generation of Circos charts specifically dedicated to epilipidomics data.^44^ In particular, LipidCircos intends to display and highlight relationships between identified epilipids and their corresponding native precursors, as well as overlaying customized information such as quantification data across samples and/or time points.^44^ In the publication, the authors showcased the package capabilities by generating a Circos plot to display oxidized phospholipids and their corresponding native species, identified in rat primary cardiomyocytes treated with peroxynitrite donor SIN-1 over 16 h (Figure 4B).

## Current GUI-Based Computational Tools to Address Epilipidomics Challenges

Several freely available tools allow the processing and analysis of LC-MS (epi)lipidomics data. Various R^90−92^ and Python^93−95^ packages have been developed in the past few years and constitute an important resource for epilipidomics applications. The majority of these packages provide a command-line interface only, which require researchers to be comfortable with coding for proper use. However, a common reality is that most lipidomics researchers lack even basic programming skills, and this limits a wide adoption of these tools by the community.^56^ For this reason, we focused the present review on prepackaged software with a user-friendly graphical user interface (GUI) as they represent a more accessible solution for researchers lacking coding expertise.

Our literature survey retrieved a total of six GUI-based software suitable to address various stages of the LC-MS epilipidomics data analysis workflow: Lipid Data Analyzer (LDA),^34,96^ Lipostar,^60^ LipidMatch Flow^97^ (recently introduced as an update to the LipidMatch R package^91^), LPPtiger 2,^44^ MS-DIAL 4,^98^ and MZmine 3.^99^ In addition, although not a data processing software per se, LipidLynxX^40^ was also included in the review as it is one of the lipid nomenclature harmonization tools, which has already integrated specific optimization for epilipids. Lipostar is proprietary but free for nonprofit institutions, while the other reviewed software packages are open source and freely available. The usability of these software for LC-MS-based epilipidomics data analysis is summarized in Table 1 and discussed below. In particular, the table highlights the data analysis steps that can be performed, along with the above-mentioned five challenges that can be addressed (at least partially) by each software.

**Table 1:** GUI-Based Software Currently Available for the Analysis of LC-MS Epilipidomics Data^a^

Software Package	Epilipidomics Data Analysis Steps	Challenges Addressed
**LDA**	*Feature detection*: Yes	1, 2, 3, 4
	*Lipid identification*: Yes	
	-*ID based on*: RT, IP, MS/MS	
	-*Highest annotation level*: *sn*-position	
	*Lipid quantification*: Yes	
	*Results investigation*: No	
**LipidLynxX**	*Feature detection*: N/A	5
	*Lipid identification*: N/A	
	*Lipid quantification*: N/A	
	*Results investigation*: N/A	
**LipidMatch Flow**	*Feature detection*: Yes (through MZmine 2)	1^b^, 2, 3, 4
	*Lipid identification:* Yes	
	-*ID based on*: MS/MS	
	-*Highest annotation level:* molecular species	
	*Lipid quantification*: Yes	
	*Results investigation*: No	
**Lipostar**	*Feature detection*: Yes	1, 2, 3, 4, 5^c^
	*Lipid identification*: Yes	
	-*ID based on*: RT, IP, MS/MS, CCS	
	-*Highest annotation level*: molecular species	
	*Quantification*: Yes	
	*Results investigation*: Yes (KMD plot)	
**LPPtiger 2**	*Feature detection*: No	2, 3, 4
	*Lipid identification*: Yes	
	-*ID based on*: RT, IP, MS/MS	
	-*Highest annotation level*: molecular species	
	*Lipid quantification*: No	
	*Results investigation*: No	
**MS-DIAL 4**	*Feature detection*: Yes	1, 2, 3, 4
	*Lipid identification*: Yes	
	-ID based on: RT, IP, MS/MS, CCS	
	-Highest *annotation level*: *sn*-position	
	*Lipid quantification*: Yes	
	*Results investigation*: No	
**MZmine 3**	*Feature detection*: Yes	1,2,3,4
	*Lipid identification*: Yes	
	-*ID based on*: RT, MS/MS, CCS	
	-*Highest annotation level*: molecular species	
	*Lipid quantification*: Yes	
	*Results investigation*: Yes (KMD plot)	

a The table summarizes the steps of the epilipidomics data analysis workflow that can be performed by each software, i.e., feature detection, lipid identification, lipid quantification, results investigation (KMD and Circos plot). Abbreviations used: ID, identification; RT, retention time; CCS, collision cross-section; IP, isotopic pattern; MS/MS, fragmentation data. The computational-related challenges that can be fully, or partially, addressed by each software are listed as follows: 1, low abundance; 2, high structural diversity; 3, high number of isomers/isobars; 4, different fragmentation compared to native lipids; 5, nomenclature.

b LipidMatch Flow relies on MZmine 2 for the feature detection and quantification.^91,97^

c Lipostar can be integrated with LipidLynxX to address (epi)lipids nomenclature. (Software are listed in alphabetical order.)

Although five of the tools listed in Table 1 (LDA, Lipostar, LipidMatch Flow, MS-DIAL 4, and MZmine 3) were not specifically designed for epilipidomics applications, they provide the user with a range of algorithms to cover various steps of the data analysis workflow shown in Figure 3, as well as the flexibility to finely tune most of the processing parameters. In contrast, LPPtiger 2 was explicitly developed for the identification of oxidized lipid species; oxidized phospholipids (oxPLs), diacylglycerols (oxDG), triacylglycerols (oxTG), and cholesteryl esters (oxCE) are supported at the time of writing.

### Dealing with Low Abundance

As explained above, epilipids often produce signals close to the noise level due to their natural low abundance. Tunable feature detection algorithms can reduce the risk of erroneously discarding relevant signals while ensuring a reasonable computation burden. Four of the software listed in Table 1 (LDA, Lipostar, MS-DIAL 4, and MZmine 3) include standalone algorithms for feature detection and allow the user to adjust the processing parameters toward low-abundance signals. In contrast, LipidMatch Flow does not provide a built-in peak picking algorithm and relies on MZmine 2^63^ for the feature detection.^91,97^ A different approach is used by LPPtiger 2, which does not require a prior feature detection to be carried out. Instead, the software predicts oxidized lipid species starting from a user-provided list of unmodified molecules of interest and searches for the corresponding MS/MS spectra directly in the raw data.

During the feature detection process, there are a number of situations where a feature might not be detected in one (or more) samples even though an LC peak is actually present. In these cases, a zero-intensity value is (erroneously) assigned, producing so-called “missing values” in the aligned feature table.^100^ This is generally caused by suboptimal parameter settings, such as overly high noise threshold, inconsistent chromatogram resolving, misalignment, etc. In this context, Lipostar, MS-DIAL 4, and MZmine 3 offer the possibility to automatically reinspect the aligned feature table to cope with false missing values that are artifacts of the processing.^60,98,99^ In these tools, the algorithm examines each missing value in the feature table individually and checks for the presence of omitted chromatographic signals in the original raw data where the peak is expected (i.e., RT window associated with the examined feature). If a meaningful LC peak is found, it is integrated and the retrieved peak area used. This approach (often referred to as “secondary feature detection” or “gap-filling”) can significantly reduce the presence of missing values in the final feature table, providing more accurate quantification data suitable for a robust downstream statistical analysis.^99^

### Dealing with High Structural Diversity, Native Lipid Isobars/Isomers, Unique Fragmentation

The epilipidome structural diversity, the frequent occurrence of isobaric/isomeric species, and the specific fragmentation patterns of certain modified lipid classes are all hurdles that directly hamper the annotation of both known and unknown (epi)lipids. For the sake of convenience and to avoid redundancies in the text, these challenges will be discussed together in this section.

As discussed in Lipid Identification, a tentative chemical structure is assigned to the detected features based on the collected MS/MS data. Annotation based on spectral matching (i.e., experimental spectra matched against a reference MS/MS library) represents the most popular approach in MS-based lipidomics^16^ and is offered by all the reviewed software, except LDA, which uses an approach based on customizable mass lists. The reference MS/MS library can be already integrated in the software package, in house-curated by the user, or generated *in silico* using a rule-based approach. Concerning the latter, class-specific fragmentation rules formulated from experimental data are considered in the generation of the *in silico* MS/MS spectra. All tools come with a set of class-specific fragmentation rules that can be variably customized by the user (i.e., LDA, MZmine 3, and Lipostar allow the user to modify and/or add new fragmentation criteria through the GUI). Intensity-free spectra can also be computed by MZmine 3 and LPPtiger 2, while intensity relationships between fragments can be defined in LDA and MS-DIAL 4 and may help in assigning the *sn-*position of acyl chains. Lipostar also generates intensity-free spectra but allows the user to associate weights to the fragment ions and take them into account for the spectral match scoring.

RT- and CCS-based constraints can be included in the identification workflow to raise the annotation confidence.^22^ MZmine 3, MS-DIAL 4, Lipostar, and LDA enable the assignment of specific RT windows to the different lipid classes, which can be used to avoid incorrect annotations. Essentially, features annotated as belonging to a certain lipid class, but eluting outside the suspected window, are discarded. CCS-based annotation constraints can be defined in MS-DIAL 4 and Lipostar. RT- and CCS-based filtering options are not available in LPPtiger 2 and LipidMatch Flow. Notably, MZmine 3 also allows discarding noisy features and/or false annotation based on the chromatographic peak shape (e.g., tailing factor) and additional filters (e.g., Kendrick mass defect).^85^ The differences in the annotation workflow customizability among the reviewed software are summarized in Table 2.

**Table 2:** Customizability of the Rule-Based Annotation Workflow Adopted by the Different Software^a^

	MS-DIAL 4	MZmine 3	Lipostar	LPPtiger 2	LDA	LipidMatch Flow
*In house* curation of MS/MS libraries	√	√	√	**–**	**–**b	**–**
*In silico* generation of MS/MS libraries	√	√	√	√	**–**b	**–**
Class-specific fragmentation rules	√	√	√	√	√	√
Customizable fragmentation rules	**–**	√	√	**–**	√	√
Visualization of MS/MS spectra	√	√	√	√	√	**–**
Annotation filters (RT, CCS, etc.)	√	√	√	**–**	√	**–**

a The functionalities provided by each software are indicated by a black check mark.

b LDA does not use MS/MS libraries, but uses user extendable mass lists for identification.

Modified lipids are currently underrepresented in reference spectral libraries (oxPLs and oxTGs represent the only epilipid classes covered in public reference MS/MS libraries at the time of writing). Therefore, some packages (i.e., Lipostar and LPPtiger 2) also offer the possibility of generating *in silico* MS/MS spectra starting from a user-provided database of lipid chemical structures.

**Figure 5:**
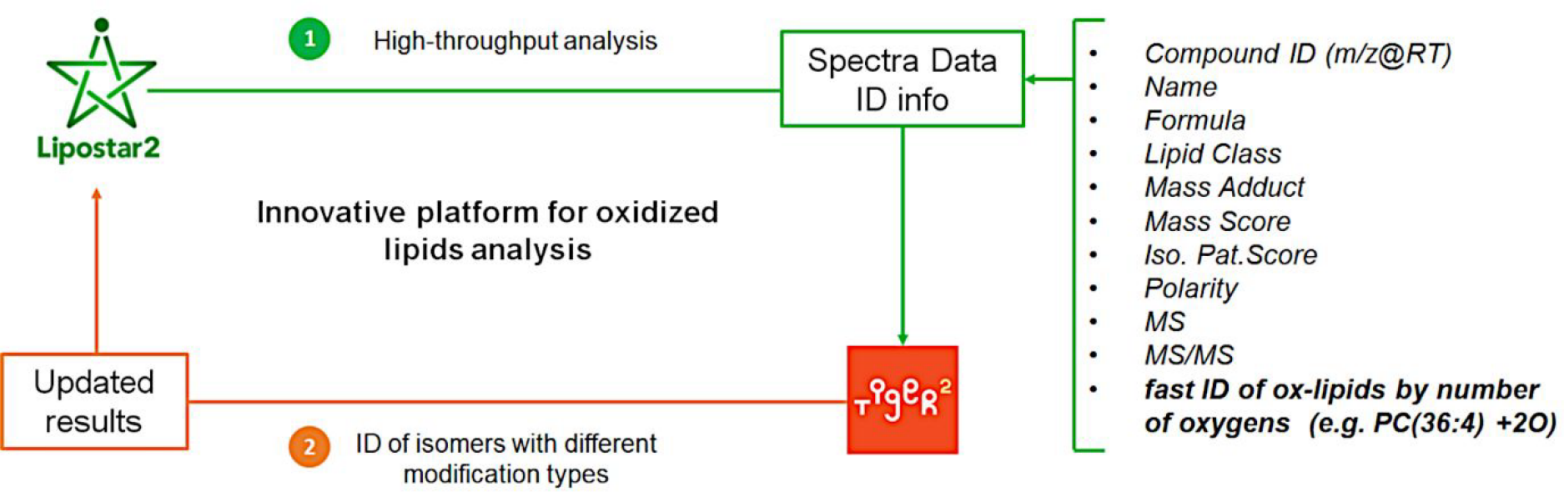
Scheme of the integration between Lipostar and LPPtiger 2.

Notably, Lipostar provides an alternative annotation approach that aims at overcoming the lack of well-characterized fragmentation rules for the majority of epilipid classes. In particular, following a first identification of native unmodified lipids via spectral matching, potential oxidized species are searched among the features in the data set that remained unannotated. The software starts from the database of native lipids structures and calculates the theoretical exact mass of the oxidized forms based on a list of user-specified modifications (e.g., M + O, M + O – 2H, etc.) and adduct type(s). Subsequently, the unknown features’ *m*/*z* are matched against the calculated oxidized lipid theoretical masses. When a match is found, the MS/MS spectrum is checked, and fragment ions exhibiting the same shift as the native-oxidized forms are taken into account in the spectral match. Moreover, a direct connection between LPPtiger 2 and Lipostar has been recently established with the aim of exploiting the benefits of both software (see Figure 5). Specifically, Lipostar can be used to perform the feature detection and a “pre-identification” (based on MS and MS/MS data) of potential oxidized species in the raw data. Such information is then used by LPPtiger 2 in its multiscoring annotation workflow.^44^ By doing so, significant computation time can be saved while consistent annotation accuracy is ensured.

### Dealing with Nomenclature

Five levels of information can be used in reporting modified lipid species: (i) the “*mass shift level*”, indicating the mass shift from the parent molecule (e.g., +16 indicates a 16 Da shift in the nominal mass, due to an hydroxylation for example); (ii) the “*elemental composition level*”, pointing out the change in the elemental composition with respect to the parent molecule (e.g., the number of additional oxygens); (iii) the “*type level*”, which relates to the type of chemical modification (e.g., two OH groups or one OOH group); (iv) the “*site level*”, referring to the position of the modification site on the parent molecule; and (v) the “*stereochemistry level*”, where information about stereochemistry is provided (e.g., 15R or 15S for OH on HETE). As discussed above, although guidelines for the shorthand reporting of modified lipid species have been recently introduced,^38^ different styles and information levels are used by the reviewed software to annotate the same species and/or modification. For instance, LDA can annotate oxidized species at the “*type level*” or the *sn*-position and the software reports them with an “ox” prefix. However, no specific nomenclature is used to discriminate between oxo, keto, epoxy, and furan modification (i.e., they are all reported as “–O–”). Lipostar and MZmine 3 provide annotation at the “*elemental composition level*” (e.g., PC 34:2 + 2O). If the specific acyl chain that underwent the oxidation can be determined from the MS/MS data, Lipostar reports the information in the identification summary. LipidMatch Flow uses an “*elemental composition level*” annotation and can specify the FA residue(s) object of the modification; three different annotation styles are available: OxPC(16:0_18:2(2O)), PC 16:0_18:2;2O, and OxTG(14:0_14:0_18:2(OHOH)). Finally, LPPtiger 2 reports the type of modification (“*type level*” annotation, e.g., PC(16:0_18:2<2OH>). Such inconsistency in reporting epilipid species limits the establishment of automated pipelines for parsing and querying lipid names across different resources (e.g., databases, software), reduces the usability of reference data, and leads to over-reporting in research papers. In this context, the recently developed LipidLynxX software aims at providing a unified nomenclature system compatible with different identification levels and that is easy-to-understand by both researchers and computer scripts. The epilipids nomenclature scheme proposed by LipidLynxX relies on a multitiered identification matrix reported in Table 3. Multiple representations of the same lipid species are avoided by means of a controlled vocabulary and an ordered list of modifications. Different abbreviation styles can be interpreted and converted into a shorthand-compatible nomenclature (e.g., Lipostar can be linked to LipidLynxX for automatic nomenclature conversion). Finally, LipidLynxX also provides a linker module to cross-link lipid abbreviations to a collection of available online databases.

**Table 3:** Multilevel Identification Matrix Used by LipidLynxX^a^

Bulk Level (B)	Molecular Species Level (M)	*sn*-Specific Level (S)
B0 = PE(36:4)	M0 = PE(16:0_20:4)	S0 = PE(16:0/20:4)
B1 = PE(36:4<+46>)	M1 = PE(16:0_20:4<+46>)	S1 = PE(16:0/20:4<+46>)
B2 = PE(36:4<+3O,–2H>)	M2 = PE(16:0_20:4<+3O,–2H>)	S2 = PE(16:0/20:4<+3O,–2H>)
B3 = PE(36:4<2OH,oxo>)	M3 = PE(16:0_20:4<2OH,oxo>)	S3 = PE(16:0/20:4<2OH,oxo>)
	M4 = PE(16:0_20:4<2OH{5,6},oxo{15}>)	S4 = PE(16:0/20:4<2OH{5,6},oxo{15}>)
	M5 = PE(16:0_20:4<2OH{5S,6R},oxo{15}>)	S5 = PE(16:0/20:4<2OH{5S,6R},oxo{15}>)

a The software combines three shorthand nomenclatures (B = bulk level; M = molecular species level; S = *sn*-specific level) with five levels of modification information (0 = no modification; 1 = modification mass shift; 2 = modification elemental composition; 3 = modification type; 4 = modification position; 5 = modification stereochemistry) into a combined matrix (e.g., B2, M3). The majority of modified lipid abbreviations can be assigned using this matrix. As an example, the M3 annotation level (i.e., molecular species + modification type) PE(16:0_20:4<2OH,oxo>) indicates that the modification type is known, but its position on the FA residues is not. The matrix can be further extended with *sn* position and modification position information, leading to the highest annotation level (i.e., S5). The double bond position and cis/trans information can be also added to further extend it to sublevels (i.e, S5.1 level: PE(16:0/20:4<{7,9,11,13}, 2OH{5S,6R},oxo{15}>) and S5.2 level PE(16:0/20:4<{7E,9E,11Z,13E},2OH{5S,6R},oxo{15}>)).

## Databases Containing Epilipids

Databases normally play a central role in any computational method and/or workflow. In this section, we review those databases that currently contain relevant information regarding epilipids.

Generic metabolite databases such as METLIN,^101^ MassBank,^102^ and the MassBank of North America (MoNA)^103^ constitute a valuable resource to assist (epi)lipid annotation as they contain both experimental and *in silico-*generated MS/MS data for a number of oxidized lipids. Other generic databases such as ChEBI^104^ and PubChem^105^ contain some epilipids but were not optimized to filter or search for epilipids specifically, however, cross-linking from other lipid databases (e.g., LIPID MAPS LMSD,^32,106^ SwissLipids^107^) is available for data integration. A list of databases where structures of epilipids are collected and easily accessible is provided in Table 4.

**Table 4:** Molecular Structure Databases That Include Modified Lipids

Database	ref	URL	Downloadable	Format	MS/MS	Annotation Levels^a^
CMM 3.0	(110)	http://ceumass.eps.uspceu.es/	No		from HMDB	type
HMDB	(107)	https://hmdb.ca	Yes	sdf	Yes	type, site, R/S
LipidBank	(109)	http://www.lipidbank.jp	No			type, site
LIPID MAPS	(32)	https://www.lipidmaps.org/databases	Yes	sdf	Yes	type, site, R/S
LipidPedia	(108)	https://lipidpedia.cmdm.tw/	No			type, site, R/S
RIKEN IMS oxidized phospholipids	(31)	http://prime.psc.riken.jp/compms/msdial/main.html#MSP	Yes	msp	Yes	type, site, R/S
SwissLipids	(105)	https://www.swisslipids.org	Yes	tsv	No	type, site, R/S

a Annotation levels: “type”, the modification type level, e.g, two OH; “site”, the modification site level, e.g, OH at position 5 and 12; “R/S”, the modification stereochemistry level, e.g, one OH on position 5 is 5R and another OH on position 12 is 12S.

LIPID MAPS (which stands for LIPID Metabolites and Pathways Strategy) is arguably the most complete and widely used gateway for lipidomics.^32,106,108^ Established in 2003, it includes databases of known lipid chemical structures (LMSD),^32^*in silico*-generated structures (i.e., LIPID MAPS In-Silico Structure Database), computationally generated oxidized phospholipid species (including the corresponding precursor ion’s *m*/*z*), experimental CCS values (i.e., LIPID MAPS Ion Mobility Database), as well as a repository of experimental lipidomics data sets.^108^ Notably, the LMSD also contains nomenclature, references, cross-links to other databases, and experimental MS/MS spectra for several lipid species. At the time of writing, the LMSD includes over 250 oxPLs, 1000 octadecanoids, 1200 eicosanoids, and 1100 docosanoids (accessed September 2022).

SwissLipids is a curated collection of known lipid structures and related information about metabolism, interactions, as well as subcellular and tissular localization.^107^ All the information is curated from peer-reviewed literature. The set of known lipid structures is also complemented by a library of theoretical structures obtained by the combination of known building blocks from the curated set. Both known and theoretical lipids are organized into a single common hierarchy that follows the notation for mass spectrometry-based lipidomic data proposed by Liebisch et al.^37,38^ and is consistent with the classification developed by LIPID MAPS. Concerning modified lipids, a collection of octadecanoids (13), eicosanoids (136), and docosanoids (13) is included, and a few complex epilipids are included in the database at the time of writing (accessed September 2022).

The Human Metabolome Database (HMDB) is a freely accessible database containing information about small molecule metabolites found in the human body.^109^ It includes a large number of both confirmed and predicted lipid structures. At the time of writing, the HMDB includes 480 eicosanoids species and over 1000 predicted oxPLs.

The Riken IMS oxidized phospholipid database contains the MS/MS spectra acquired in negative ion mode of 386 total molecular species of oxPLs obtained by biogenic conversion from oxidized FAs incorporated into cellular phospholipids. More details can be found in Aoyagi et al.^31^

Notably, LIPID MAPS LMSD, SwissLipids, RIKEN IMS oxidized phospholipids, and HMDB are downloadable and can be conveniently connected to different software packages to assist the (epi)lipid annotation pipeline (e.g., Lipostar allows the direct import of the LMSD).^60^

Other lipid structure databases (e.g., LipidPedia,^110^ LipidBank^111^ or CEU mass mediator^112^) are available only online. Notably, CEU mass mediator 3.0 (CMM 3.0) integrates compounds from different sources (e.g., HMDB, LIPID MAPS, KEGG, Metlin, etc.) and offers a module to annotate oxidized lipids.^112^ LipidBank is the official database of the Japanese Conference on the Biochemistry of Lipids and contains molecular structures, spectral data (MS, NMR, etc.), and literature information for more than 7000 unique lipid species. Among them, 329 eicosanoids and 5 lipid peroxides are present (accessed September 2022). LipidPedia is a database of biomedical information for over 4400 lipid species retrieved from the literature using text-mining strategies with more than 1,500,000 associated literature references (accessed September 2022). At the time of writing, searching the term “oxidised” in the database returns only 6 six entries, although comprehensive information (e.g., classification, biological functions, biomedical data, etc.) is provided.

## Emerging Tools for Epilipidomics

In this section we review emerging computational strategies that can further assist the analysis and annotation of epilipidomics LC-MS data. These methods were originally developed for different applications (e.g., computational biology or metabolomics) but are finding a wider use in epilipidomics studies.

### Metabolic Network Analysis

Metabolic network analysis is a powerful visualization tool widely used in computational biology. It can be used to display qualitative and quantitative changes in the lipids profile at a system level. A metric particularly interesting for epilipidomics applications is the change of (partial) correlations between pairs of species under different experimental conditions. The intuition here is that a reaction connecting two (epi)lipid species is more likely to be active when the respective epilipids are highly correlated. Consequently, a loss of high correlation between experimental conditions is a hint toward changes in reaction activity. Similar ideas have already been shown to generate novel insights into the regulation of epilipid biosynthesis. Lauder et al.,^113^ for example, found specific lipoxygenases to be important for coagulation and the thrombotic disorder antiphospholipid syndrome (APS) using an epilipid correlation-network approach.

One lipidomics-specific method for extensive network analysis is the Lipid Network Explorer,^114,115^ abbreviated as LINEX. It is based on networks that combine fatty acid and lipid class metabolic reactions to compute species-level biochemical connections. Due to this modular structure, it is well-suited to cover epilipids by incorporating position-specific hydroxylation patterns and reactions.

To enable a quantitative analysis of lipidomics results, LINEX utilizes statistical metrics such as fold-changes and *p*-values (between sample groups) as well as correlation metrics (between lipid species, per sample group). By projecting differences in sample groups onto the network, it allows the exploration of global changes in the lipidome. Since the network topology entirely depends on lipid metabolic reactions, this enables the identification of groups of reactions with distinct patterns between sample groups as well as generating hypotheses on alterations in enzymatic activity.

Epilipid metabolism pathways are partially searchable in databases (such as Rhea^116^ and Reactome^117^), and it is now possible to generate reasonable epilipidome metabolic networks. Nevertheless, additional efforts should be made to integrate the available information, enable the application of active module identification algorithms, and set the basis for metabolic modeling of the epilipidome. Furthermore, such networks will enable the integration of epilipidomics data with data from proteomics and other omics disciplines. We can therefore expect epilipidomics data interpretation to deliver novel insights into disease mechanisms and general biological processes.

### Molecular Networking

Molecular networking (MN) is a relatively new computational strategy for the analysis and visualization of untargeted LC-MS data.^118^ It relies on the fundamental assumption that molecules sharing similarities in their chemical structures also share fragment ion patterns when subjected to MS/MS fragmentation methods. Building on this hypothesis, MN creates networks of MS/MS spectral relations where structurally related molecules are connected together. Briefly, each MS/MS spectrum in a data set is compared pairwise against every other, and the spectral similarity is assessed using a modified cosine similarity score. Here, not only signals at identical *m*/*z* are taken into account, but also fragment ions that are offset by the same *m*/*z* difference as the precursors are considered as “matching” peaks.^119^ Based on the calculated similarity scores, MS/MS data are organized into a network where each ion is represented as a node, and ions sharing an MS/MS spectral similarity above the user-defined threshold are connected by an edge. By doing so, molecules with similar chemical structures (e.g., differing by simple transformations such as oxidation/reduction, glycosylation, alkylation, *etc*.) appear as connected nodes and cluster into “molecular families”. This tremendously assists the visualization of the detected chemical space and greatly facilitates the annotation of unknown, structurally related metabolites within a molecular network. Moreover, in combination with common feature-finding tools, semiquantitative information (peak area) can be mapped over each node in the network to infer semiquantitative differences among samples or sample groups.^119^ Although originally developed for metabolomics data analysis to assist the identification of novel metabolites,^120^ molecular networking has recently been applied in several (epi)lipidomics studies.^121−126^ Notably, Watrous et al. applied untargeted LC-MS/MS in conjunction with MN networking to expand the repertoire of oxylipins in human plasma samples by annotating 362 putatively known oxylipins and 46 unknown related compounds.^121^ As demonstrated by this study, the epilipidomics field can greatly benefit from MN and MN-based strategies for the rapid prioritization and annotation of both known and unknown modified lipids through the network connections. Although other solutions exist,^127^ the most widely used platform to carry out MN analysis is currently the Global Natural Products Social Molecular Networking (GNPS) ecosystem,^118,119^ which offers a web-based GUI and extensive documentation resources for new users.

### Machine Learning-Based Tools

Machine learning (ML) approaches to support the untargeted LC-MS data analysis workflow are becoming more and more popular. For example, ML-assisted algorithms have been developed for the automated peak picking and filtering of noise features based on chromatographic peak shape and intensity.^128−131^ Another interesting application of ML-based methods is the prediction of RT^132^ and ion mobility-derived CCS^133^ values for metabolite identification purposes. The goal of these prediction models is the calculation of these values without the need of their experimental measurement (which require reference samples and/or standards). Notably, Zhou et al. developed a dedicated model for the prediction of CCS for lipids.^134^ A further application of ML for metabolite annotation is represented by tools for the automated interpretation of MS/MS data, such as the SIRIUS software suite^135^ and MSNovelist.^136^ In particular, these methods offer the possibility of database-free predictions of elemental formulas and chemical structures that do not restrict the annotation to searching against structural databases (e.g., PubChem, LMSD) and/or matching against experimental MS/MS spectral libraries. This can be of particular help in the identification of truly new, unreported metabolites. Notably, the recently released SIRIUS 5 includes a dedicated module for lipid structure predictions.^137^

### Mass Spectrometry Search Tool and Mass Spectrometry Query Language

Deposition of untargeted MS data in the public domain is experiencing rapid growth, largely thanks to the increasing adoption of universal, nonvendor-specific MS data formats (e.g., mzML format). The two MS data mining tools described in this section, the *Mass Spectrometry Search Tool* (MASST)^138^ and the *Mass Spectrometry Query Language* (MassQL),^139^ have been recently developed by the Dorrestein lab with the aim of making the ever-growing untargeted MS data repositories (∼12,000 data sets comprising ∼7,500,000 files as of September 2022) an easily accessible resource to assist the annotation of unknown molecules and structural analogues.

The MASST tool is a web-based search engine for MS data.^138,140^ It enables querying of MS/MS spectra across public spectral libraries (e.g., GNPS libraries,^118,140^ all three MassBanks,^102^ etc.) and repositories of metabolomics MS data (e.g., GNPS/MassIVE,^118^ Metabolomics Workbench,^141^ MetaboLights,^142^ etc.). A specific MS/MS spectrum of interest can be searched by copy–pasting the spectrum peak list and defining a set of search tolerances within a user-friendly web interface.^140^ A query result includes all matches to identical or analogous MS/MS spectra in public spectral libraries and repositories along with their associated metadata and/or sample information. In particular, metadata can be linked at the data set level (e.g., instrument type, taxonomy, keywords), file level (e.g., sample type, age, sex, body site, disease, etc.) or single annotated spectrum level (e.g., biological activity and structural class information).

MassQL is a novel query language for the mining of MS data.^139^ Inspired by the SQL programming language, MassQL implements a consensus vocabulary to search for MS patterns using human-readable query strings. Searchable MS terms include both MS (e.g., precursor ion *m*/*z*, isotopic patterns) and MS/MS fragmentation patterns (e.g., diagnostic fragments and neutral losses), with support for both data-dependent (DDA) and data-independent acquisition (DIA). Additionally, terms for separation methods (i.e., retention time and ion mobility drift time), user-defined tolerances (e.g., ion intensity, mass accuracy), and boolean conjunctions (i.e., AND, OR) can be used to define inclusion/exclusion criteria and create more complex pattern queries. As an example, choline-containing phospholipids normally exhibit diagnostic fragments and neutral losses in the MS/MS spectra (positive ionization) arising from the glycerophosphocholine group: *m*/*z* 184.0739 and corresponding neutral loss of *m*/*z* 183.0733 (phosphocholine), *m*/*z* 125.0004 (2,2-dihydroxy-1,3,2-dioxaphospholan-2-ium).^143^ The following MassQL query string can be formulated to quickly retrieve all the MS/MS spectra that contain such diagnostic fragments/losses (within a 10 ppm mass tolerance) and likely belong to glycerophosphocholine lipids:

QUERY scaninfo(MS2DATA) WHERE
MS2PROD = 184.0739:TOLERANCEPPM = 10 AND
MS2PROD = 125.0004:TOLERANCEPPM = 10 AND
MS2NL = 183.0733:TOLERANCEPPM = 10PPM

The data search can be carried out on a single file, data set, or scaled up to entire MS data repositories. As for MASST, MassQL query results include all matches to identical/analogous MS/MS spectra in spectral libraries and data repositories together with the associated metadata.

## Summary and Outlook

The growing interest in epilipidomics has boosted the development of *ad hoc* strategies for data analysis in recent years, although currently standard data analysis pipelines are missing. In this review, we first aimed at describing the available software for lipidomics endowed with a graphical user interface (to facilitate their use) and freely available for academic use. Though some workflows, which were originally developed for generic unmodified lipidomics or metabolomics, can be tweaked to analyze certain epilipids, epilipids-specific criteria still demand extra focus. From the availability of solid structure- and spectral libraries to the confidence of identification algorithms, data processing of epilipids has more challenges than well-developed metabolomics workflows. For example, due to the similarities of multiple isomeric species (e.g., two OH or one OOH) at different modification sites, the algorithms for the identification of exact epilipid structures and for accurate quantification of each isomer are required to implement multiple filters and rules that relate to specific epilipid classes and differ from common metabolomics algorithms, with the risk of becoming time-consuming. These unique challenges lead to adapting or developing computational tools for epilipidomics, which are suitable to deal with the intrinsic complexity of the epilipidome (*intrinsic challenges*) at different levels, as well as with the *analytical challenges* in the field. Our analysis brings us to believe that, although several steps forward have been made to develop software and computational approaches for epilipidomics, the currently available *in silico* tools still suffer some limitations due to experimental-derived and pure computational issues.

From the experimental perspective, the lack of a large collection of standards for epilipidomics has a negative impact both on quantification and on the accurate definition of fragmentation rules or spectral matching strategies to be used in identification. Similarly, while CCS libraries are available for native lipids, the collection of CCS values for epilipids is still in its infancy. *In silico* predicted CCS values generated by epilipid-specific algorithms might be a potential starting point, but experimental CCS values for epilipids are needed to validate predictions. Finally, the analysis of the epilipidome needs accurate analytical procedures to reduce the risk of analyzing artifacts. Indeed, potential degradation products like oxidized lipids could form during sample processing if not correctly handled, and this will hamper any software’s ability to deliver reliable results.^76^

From the computational perspective, referring to the four steps of data analysis discussed in this review, *lipid quantification* and *results investigation* will require only minor improvements in the existing software, taking advantage from the experience in the field of native lipids. For example, the main bottleneck in *lipid quantification* is again the lack of proper standards. In addition, one needs to consider the specific changes of adduct distribution for epilipids, and the use of adduct clustering algorithms already available for native lipids have not yet been extensively validated for epilipids. Therefore, we still recommend reviewing the automatic assignment of lipid identification together with peak integration to make quantification reliable. Concerning the *feature detection* step, we described several tools that facilitate peak picking close to the noise level (e.g., using the gap-filling algorithms), which will be very beneficial for epilipidomics. It is noteworthy that, while LC-MS/MS data acquired in DDA mode are widely supported, only Lipostar, LipidMatch Flow, and MS-DIAL 4 support DIA mode such as All Ion Fragmentation (AIF), MS^E^, or SWATH. About the *lipid identification* step, we believe that large improvements are still possible to deal with the high number of isomers and isobars. So far, only LPPtiger 2 was specifically designed to identify epilipids from a number of classes (several phospholipids, cholesteryl esters, diacylglycerols, and triacylglycerols) with the aim of discriminating isomers and isobars with the drawback of being a time-consuming analysis, while the other software here discussed propose faster but less accurate alternative solutions. A first attempt of making a step forward in epilipid identification is the recent link between LPPtiger 2 and Lipostar 2 that allows having the accurate annotation of epilipids of the first but in a short time thanks to a preanalysis made by Lipostar 2. Similarly, we suggest that other software could connect to LPPtiger 2 in the near future. However, at this stage we still recommend manual rechecking of automatically identified epilipids to reduce misannotations and over-reported species (due to adduct clustering failure) to overall improve the quality of epilipidomics data analysis. Indeed, taking into account the well-known issue of the lack of standards, the prediction of epilipids’ properties such as fragmentation pattern, retention time, or CCS value could partially consolidate lipid identification. Machine learning tools offer the potential to predict CCS and RT values for small molecules with high accuracy,^144,145^ but they have not been suitably explored for lipids or epilipids, where a high degree of rotatable bonds leads to a higher complexity and lower performance. Nevertheless, concerning RT prediction, elution order prediction could be preferred in a first attempt, as it provides more stable results.^146^

The scattered solutions, each with strong focus in certain fields, call for joining the efforts through collaborative workflow connecting multiple tools. In addition, there is a trend to adapt new algorithms from metabolomics including molecular networking and machine learning to epilipidomics with additional adjustments. However, unlike several algorithms that can be applied to a large number of different metabolite classes across highly diverse structures,^147^ it is still challenging to develop algorithms that can correctly distinguish epilipid isomers with high confidence.

Finally, currently available databases containing information about epilipids were also described. Databases containing structures of epilipids and/or related MS/MS libraries are still very limited and fragmented. Among the various sources, searching for the number of epilipids considered in a given database is still a challenging task, probably due to the limited attention that has been devoted in the past to epilipids. The lack of well-defined keywords, labeling systems, and, *in primis*, a common nomenclature, hampers the effort of collecting the available information. The use of a standard nomenclature is essential for sharing and comparing lipid annotations and to browse into databases. LipidLynxX has the merit of being a tool able to convert different annotations to unified identifiers based on a community-accepted shorthand notation system.^37,38^ The computational community should work in connecting software to this tool to have a common nomenclature system (software such as BioPAN,^148^ LINEX, Lipostar 2, and LPPtiger 2 are already linked to LipidLynxX). Finally, an increase of the number of epilipid species in databases will offer new perspectives for computational approaches, although the limited number of standards for epilipidomics hampers the construction of large databases of experimental data.

To conclude, we must emphasize that much of what is shown and discussed in this review is related to oxidized lipids only, which represent only a part of the epilipidome. The new frontier, therefore, will be to broadly investigate the epilipidome space in its entirety and complexity and to develop new *in silico* tools dealing with new and emerging modified lipids in complex matrices by applying untargeted approaches.

## References

[ref1] Snaebjornsson M. T.; Janaki-Raman S.; Schulze A. Greasing the Wheels of the Cancer Machine: The Role of Lipid Metabolism in Cancer. Cell Metab 2020, 31 (1), 62–76. 10.1016/j.cmet.2019.11.010. 31813823

[ref2] Nagao K.; Yanagita T. Functional Lipids in Metabolic Syndrome. J. Nutr Sci. Vitaminol (Tokyo) 2015, 61 (Supplement), S159–S161. 10.3177/jnsv.61.S159. 26598838

[ref3] Jeong D.-W.; Lee S.; Chun Y.-S. How Cancer Cells Remodel Lipid Metabolism: Strategies Targeting Transcription Factors. Lipids Health Dis 2021, 20 (1), 163 10.1186/s12944-021-01593-8. 34775964 PMC8590761

[ref4] Yost H.; Patel R.; Kipp A.; Reed T. T. Lipids in Neurodegenerative Diseases. In Bailey’s Industrial Oil and Fat Products; Shahidi F., Ed.; John Wiley & Sons, Ltd, 2020; pp 1–29 10.1002/047167849X.BIO115.

[ref5] Freigang S. The Regulation of Inflammation by Oxidized Phospholipids. Eur. J. Immunol. 2016, 46 (8), 1818–1825. 10.1002/eji.201545676. 27312261

[ref6] Melo T.; Montero-Bullón J.-F.; Domingues P.; Domingues M. R. Discovery of Bioactive Nitrated Lipids and Nitro-Lipid-Protein Adducts Using Mass Spectrometry-Based Approaches. Redox Biol. 2019, 23, 101106 10.1016/j.redox.2019.101106. 30718106 PMC6859590

[ref7] Spickett C. M. Chlorinated Lipids and Fatty Acids: An Emerging Role in Pathology. Pharmacol Ther 2007, 115 (3), 400–409. 10.1016/j.pharmthera.2007.06.002. 17658610

[ref8] Dennis E. A.; Norris P. C. Eicosanoid Storm in Infection and Inflammation. Nat. Rev. Immunol 2015, 15 (8), 511–523. 10.1038/nri3859. 26139350 PMC4606863

[ref9] O’Donnell V. B.; Aldrovandi M.; Murphy R. C.; Krönke G. Enzymatically Oxidized Phospholipids Assume Center Stage as Essential Regulators of Innate Immunity and Cell Death. Sci. Signal 2019, 12 (574), eaau2293 10.1126/scisignal.aau2293. 30914483

[ref10] Buchan G. J.; Bonacci G.; Fazzari M.; Salvatore S. R.; Gelhaus Wendell S. Nitro-Fatty Acid Formation and Metabolism. Nitric Oxide 2018, 79, 38–44. 10.1016/j.niox.2018.07.003. 30006146 PMC6241218

[ref11] Schopfer F. J.; Khoo N. K. H. Nitro-Fatty Acid Logistics: Formation, Biodistribution, Signaling, and Pharmacology. Trends Endocrinol Metab 2019, 30 (8), 505–519. 10.1016/j.tem.2019.04.009. 31196614 PMC7121905

[ref12] Narzt M. S.; Pils V.; Kremslehner C.; Nagelreiter I. M.; Schosserer M.; Bessonova E.; Bayer A.; Reifschneider R.; Terlecki-Zaniewicz L.; Waidhofer-Söllner P.; Mildner M.; Tschachler E.; Cavinato M.; Wedel S.; Jansen-Dürr P.; Nanic L.; Rubelj I.; El-Ghalbzouri A.; Zoratto S.; Marchetti-Deschmann M.; Grillari J.; Gruber F.; Lämmermann I. Epilipidomics of Senescent Dermal Fibroblasts Identify Lysophosphatidylcholines as Pleiotropic Senescence-Associated Secretory Phenotype (SASP) Factors. J. Invest Dermatol 2021, 141 (4S), 993–1006.e15. 10.1016/j.jid.2020.11.020. 33333126

[ref13] Sochorová M.; Vávrová K.; Fedorova M.; Ni Z.; Slenter D.; Kutmon M.; Willighagen E. L.; Letsiou S.; Töröcsik D.; Marchetti-Deschmann M.; Zoratto S.; Kremslehner C.; Gruber F. Research Techniques Made Simple: Lipidomic Analysis in Skin Research. J. Invest Dermatol 2022, 142 (1), 4–11.e1. 10.1016/j.jid.2021.09.017. 34924150

[ref14] Gruber F.; Marchetti-Deschmann M.; Kremslehner C.; Schosserer M. The Skin Epilipidome in Stress, Aging, and Inflammation. Front Endocrinol (Lausanne) 2021, 11, 607076 10.3389/fendo.2020.607076. 33551998 PMC7859619

[ref15] Neves B.; Pérez-Sala D.; Ferreira H. B.; Guerra I. M. S.; Moreira A. S. P.; Domingues P.; Domingues M. R.; Melo T. Understanding the Nitrolipidome: From Chemistry to Mass Spectrometry and Biological Significance of Modified Complex Lipids. Prog. Lipid Res. 2022, 87, 101176 10.1016/j.plipres.2022.101176. 35636567

[ref16] Ni Z.; Goracci L.; Cruciani G.; Fedorova M. Computational Solutions in Redox Lipidomics - Current Strategies and Future Perspectives. Free Radic Biol. Med. 2019, 144, 110–123. 10.1016/j.freeradbiomed.2019.04.027. 31035005

[ref17] Wang M.; Wang C.; Han R. H.; Han X. Novel Advances in Shotgun Lipidomics for Biology and Medicine. Prog. Lipid Res. 2016, 61, 83–108. 10.1016/j.plipres.2015.12.002. 26703190 PMC4733395

[ref18] Li J.; Vosegaard T.; Guo Z. Applications of Nuclear Magnetic Resonance in Lipid Analyses: An Emerging Powerful Tool for Lipidomics Studies. Prog. Lipid Res. 2017, 68, 37–56. 10.1016/j.plipres.2017.09.003. 28911967

[ref19] Jurowski K.; Kochan K.; Walczak J.; Barańska M.; Piekoszewski W.; Buszewski B. Analytical Techniques in Lipidomics: State of the Art. Crit Rev. Anal Chem. 2017, 47 (5), 418–437. 10.1080/10408347.2017.1310613. 28340309

[ref20] Li C.; Chu S.; Tan S.; Yin X.; Jiang Y.; Dai X.; Gong X.; Fang X.; Tian D. Towards Higher Sensitivity of Mass Spectrometry: A Perspective From the Mass Analyzers. Front Chem. 2021, 9, 813359 10.3389/fchem.2021.813359. 34993180 PMC8724130

[ref21] Helmer P. O.; Wienken C. M.; Korf A.; Hayen H. Mass Spectrometric Investigation of Cardiolipins and Their Oxidation Products after Two-Dimensional Heart-Cut Liquid Chromatography. J. Chromatogr A 2020, 1619, 460918 10.1016/j.chroma.2020.460918. 32008819

[ref22] Paglia G.; Smith A. J.; Astarita G. Ion Mobility Mass Spectrometry in the Omics Era: Challenges and Opportunities for Metabolomics and Lipidomics. Mass Spectrom Rev. 2022, 41 (5), 722–765. 10.1002/mas.21686. 33522625

[ref23] Dyall S. C.; Balas L.; Bazan N. G.; Brenna J. T.; Chiang N.; da Costa Souza F.; Dalli J.; Durand T.; Galano J.-M.; Lein P. J.; Serhan C. N.; Taha A. Y. Polyunsaturated Fatty Acids and Fatty Acid-Derived Lipid Mediators: Recent Advances in the Understanding of Their Biosynthesis, Structures, and Functions. Prog. Lipid Res. 2022, 86, 101165 10.1016/j.plipres.2022.101165. 35508275 PMC9346631

[ref24] Galano J. M.; Lee Y. Y.; Oger C.; Vigor C.; Vercauteren J.; Durand T.; Giera M.; Lee J. C. Y. Isoprostanes, Neuroprostanes and Phytoprostanes: An Overview of 25 Years of Research in Chemistry and Biology. Prog. Lipid Res. 2017, 68, 83–108. 10.1016/j.plipres.2017.09.004. 28923590

[ref25] Tyurina Y. Y.; Tyurin V. A.; Anthonymuthu T.; Amoscato A. A.; Sparvero L. J.; Nesterova A. M.; Baynard M. L.; Sun W.; He R. R.; Khaitovich P.; Vladimirov Y. A.; Gabrilovich D. I.; Bayır H.; Kagan V. E. Redox Lipidomics Technology: Looking for a Needle in a Haystack.. Chem. Phys. Lipids 2019, 221, 93–107. 10.1016/j.chemphyslip.2019.03.012. 30928338 PMC6714565

[ref26] Spickett C. M. Formation of Oxidatively Modified Lipids as the Basis for a Cellular Epilipidome. Front Endocrinol (Lausanne) 2020, 11, 602771 10.3389/fendo.2020.602771. 33408694 PMC7779974

[ref27] Simões C.; Silva A. C.; Domingues P.; Laranjeira P.; Paiva A.; Domingues M. R. M. Phosphatidylethanolamines Glycation, Oxidation, and Glycoxidation: Effects on Monocyte and Dendritic Cell Stimulation. Cell Biochem Biophys 2013, 66 (3), 477–487. 10.1007/s12013-012-9495-2. 23250583

[ref28] Melo T.; Silva E. M. P.; Simões C.; Domingues P.; Domingues M. R. M. Photooxidation of Glycated and Non-Glycated Phosphatidylethanolamines Monitored by Mass Spectrometry. J. Mass Spectrom 2013, 48 (1), 68–78. 10.1002/jms.3129. 23303749

[ref29] Maciel E.; Neves B. M.; Santinha D.; Reis A.; Domingues P.; Teresa Cruz M.; Pitt A. R.; Spickett C. M.; Domingues M. R. M. Detection of Phosphatidylserine with a Modified Polar Head Group in Human Keratinocytes Exposed to the Radical Generator AAPH. Arch. Biochem. Biophys. 2014, 548, 38–45. 10.1016/j.abb.2014.02.002. 24560783

[ref30] Hammond V. J.; O’Donnell V. B. Esterified Eicosanoids: Generation, Characterization and Function. Biochim. Biophys. Acta 2012, 1818 (10), 2403–2412. 10.1016/j.bbamem.2011.12.013. 22200400 PMC3740819

[ref31] Aoyagi R.; Ikeda K.; Isobe Y.; Arita M. Comprehensive Analyses of Oxidized Phospholipids Using a Measured MS/MS Spectra Library. J. Lipid Res. 2017, 58 (11), 2229–2237. 10.1194/jlr.D077123. 28874441 PMC5665662

[ref32] Sud M.; Fahy E.; Cotter D.; Brown A.; Dennis E. A.; Glass C. K.; Merrill A. H.; Murphy R. C.; Raetz C. R. H.; Russell D. W.; Subramaniam S. LMSD: LIPID MAPS Structure Database. Nucleic Acids Res. 2007, 35 (Database), D527–D532. 10.1093/nar/gkl838. 17098933 PMC1669719

[ref33] Heiles S. Advanced Tandem Mass Spectrometry in Metabolomics and Lipidomics-Methods and Applications. Anal Bioanal Chem. 2021, 413 (24), 5927–5948. 10.1007/s00216-021-03425-1. 34142202 PMC8440309

[ref34] Hartler J.; Triebl A.; Ziegl A.; Trötzmüller M.; Rechberger G. N.; Zeleznik O. A.; Zierler K. A.; Torta F.; Cazenave-Gassiot A.; Wenk M. R.; Fauland A.; Wheelock C. E.; Armando A. M.; Quehenberger O.; Zhang Q.; Wakelam M. J. O.; Haemmerle G.; Spener F.; Köfeler H. C.; Thallinger G. G. Deciphering Lipid Structures Based on Platform-Independent Decision Rules. Nat. Methods 2017, 14 (12), 1171–1174. 10.1038/nmeth.4470. 29058722 PMC5988032

[ref35] Kind T.; Tsugawa H.; Cajka T.; Ma Y.; Lai Z.; Mehta S. S.; Wohlgemuth G.; Barupal D. K.; Showalter M. R.; Arita M.; Fiehn O. Identification of Small Molecules Using Accurate Mass MS/MS Search. Mass Spectrom Rev. 2018, 37 (4), 513–532. 10.1002/mas.21535. 28436590 PMC8106966

[ref36] Oliw E. H.; Stark K.; Bylund J. Oxidation of Prostaglandin H2 and Prostaglandin H2 Analogues by Human Cytochromes P450: Analysis of ω-Side Chain Hydroxy Metabolites and Four Steroisomers of 5-Hydroxyprostaglandin I1 by Mass Spectrometry. Biochem. Pharmacol. 2001, 62 (4), 407–415. 10.1016/S0006-2952(01)00683-9. 11448449

[ref37] Liebisch G.; Vizcaíno J. A.; Köfeler H.; Trötzmüller M.; Griffiths W. J.; Schmitz G.; Spener F.; Wakelam M. J. O. Shorthand Notation for Lipid Structures Derived from Mass Spectrometry. J. Lipid Res. 2013, 54 (6), 1523–1530. 10.1194/jlr.M033506. 23549332 PMC3646453

[ref38] Liebisch G.; Fahy E.; Aoki J.; Dennis E. A.; Durand T.; Ejsing C. S.; Fedorova M.; Feussner I.; Griffiths W. J.; Köfeler H.; Merrill A. H.; Murphy R. C.; O’Donnell V. B.; Oskolkova O.; Subramaniam S.; Wakelam M. J. O.; Spener F. Update on LIPID MAPS Classification, Nomenclature, and Shorthand Notation for MS-Derived Lipid Structures. J. Lipid Res. 2020, 61 (12), 1539–1555. 10.1194/jlr.S120001025. 33037133 PMC7707175

[ref39] O’Donnell V. B.; FitzGerald G. A.; Murphy R. C.; Liebisch G.; Dennis E. A.; Quehenberger O.; Subramaniam S.; Wakelam M. J. O. Steps Toward Minimal Reporting Standards for Lipidomics Mass Spectrometry in Biomedical Research Publications. Circ Genom Precis Med. 2020, 13 (6), e003019 10.1161/CIRCGEN.120.003019. 33196315 PMC8376269

[ref40] Ni Z.; Fedorova M. LipidLynxX: A Data Transfer Hub to Support Integration of Large Scale Lipidomics Datasets. bioRxiv 2020, 2020.04.09.033894 10.1101/2020.04.09.033894.

[ref41] Fahy E.; Subramaniam S.; Brown H. A.; Glass C. K.; Merrill A. H.; Murphy R. C.; Raetz C. R. H.; Russell D. W.; Seyama Y.; Shaw W.; Shimizu T.; Spener F.; van Meer G.; VanNieuwenhze M. S.; White S. H.; Witztum J. L.; Dennis E. A. A Comprehensive Classification System for Lipids. J. Lipid Res. 2005, 46 (5), 839–861. 10.1194/jlr.E400004-JLR200. 15722563

[ref42] Fahy E.; Subramaniam S.; Murphy R. C.; Nishijima M.; Raetz C. R. H.; Shimizu T.; Spener F.; van Meer G.; Wakelam M. J. O.; Dennis E. A. Update of the LIPID MAPS Comprehensive Classification System for Lipids. J. Lipid Res. 2009, 50 (Suppl), S9–S14. 10.1194/jlr.R800095-JLR200. 19098281 PMC2674711

[ref43] Liebisch G.; Ahrends R.; Arita M.; Arita M.; Bowden J. A.; Ejsing C. S.; Griffiths W. J.; Holčapek M.; Köfeler H.; Mitchell T. W.; Wenk M. R.; Ekroos K. Lipidomics Needs More Standardization. Nat. Metab 2019, 1 (8), 745–747. 10.1038/s42255-019-0094-z. 32694765

[ref44] Ni Z.; Angelidou G.; Hoffmann R.; Fedorova M. LPPtiger Software for Lipidome-Specific Prediction and Identification of Oxidized Phospholipids from LC-MS Datasets. Sci. Rep 2017, 7 (1), 15138 10.1038/s41598-017-15363-z. 29123162 PMC5680299

[ref45] Qiao L.; Ge A.; Liang Y.; Ye S. Oxidative Degradation of the Monolayer of 1-Palmitoyl-2-Oleoyl-Sn-Glycero-3-Phosphocholine (POPC) in Low-Level Ozone. J. Phys. Chem. B 2015, 119 (44), 14188–14199. 10.1021/acs.jpcb.5b08985. 26463524

[ref46] Milic I.; Fedorova M. Derivatization and Detection of Small Aliphatic and Lipid-Bound Carbonylated Lipid Peroxidation Products by ESI-MS. Methods Mol. Biol. 2015, 1208, 3–20. 10.1007/978-1-4939-1441-8_1. 25323495

[ref47] Afonso C. B.; Spickett C. M. Lipoproteins as Targets and Markers of Lipoxidation. Redox Biol. 2019, 23, 101066 10.1016/j.redox.2018.101066. 30579928 PMC6859580

[ref48] Fruhwirth G. O.; Loidl A.; Hermetter A. Oxidized Phospholipids: From Molecular Properties to Disease. Biochim. Biophys. Acta 2007, 1772 (7), 718–736. 10.1016/j.bbadis.2007.04.009. 17570293

[ref49] Code C.; Mahalka A. K.; Bry K.; Kinnunen P. K. J. Activation of Phospholipase A2 by 1-Palmitoyl-2-(9’-Oxo-Nonanoyl)-Sn-Glycero-3-Phosphocholine in Vitro. Biochim. Biophys. Acta 2010, 1798 (8), 1593–1600. 10.1016/j.bbamem.2010.05.002. 20462500

[ref50] Kopczynski D.; Hoffmann N.; Peng B.; Ahrends R. Goslin: A Grammar of Succinct Lipid Nomenclature. Anal. Chem. 2020, 92 (16), 10957–10960. 10.1021/acs.analchem.0c01690. 32589019 PMC7467413

[ref51] Boyce J. A. The Role of 15 Lipoxygenase 1 in Asthma Comes into Focus. J. Clin Invest 2022, 132 (1), e155884 10.1172/JCI155884. 34981786 PMC8718133

[ref52] Luo Y.; Jin M.; Lou L.; Yang S.; Li C.; Li X.; Zhou M.; Cai C. Role of Arachidonic Acid Lipoxygenase Pathway in Asthma. Prostaglandins Other Lipid Mediat 2022, 158, 106609 10.1016/j.prostaglandins.2021.106609. 34954219

[ref53] Jiang W.; Jin Y.; Zhang S.; Ding Y.; Huo K.; Yang J.; Zhao L.; Nian B.; Zhong T. P.; Lu W.; Zhang H.; Cao X.; Shah K. M.; Wang N.; Liu M.; Luo J. PGE2 Activates EP4 in Subchondral Bone Osteoclasts to Regulate Osteoarthritis. Bone Res. 2022, 10 (1), 27 10.1038/s41413-022-00201-4. 35260562 PMC8904489

[ref54] Melo T.; Domingues P.; Ferreira R.; Milic I.; Fedorova M.; Santos S. M.; Segundo M. A.; Domingues M. R. M. Recent Advances on Mass Spectrometry Analysis of Nitrated Phospholipids. Anal. Chem. 2016, 88 (5), 2622–2629. 10.1021/acs.analchem.5b03407. 26814598

[ref55] Villaseñor A.; Godzien J.; Barker-Tejeda T. C.; Gonzalez-Riano C.; López-López Á.; Dudzik D.; Gradillas A.; Barbas C. Analytical Approaches for Studying Oxygenated Lipids in the Search of Potential Biomarkers by LC-MS. Trends Analyt Chem. 2021, 143, 116367 10.1016/j.trac.2021.116367.

[ref56] Chang H. Y.; Colby S. M.; Du X.; Gomez J. D.; Helf M. J.; Kechris K.; Kirkpatrick C. R.; Li S.; Patti G. J.; Renslow R. S.; Subramaniam S.; Verma M.; Xia J.; Young J. D. A Practical Guide to Metabolomics Software Development. Anal. Chem. 2021, 93 (4), 1912–1923. 10.1021/acs.analchem.0c03581. 33467846 PMC7859930

[ref57] Tautenhahn R.; Böttcher C.; Neumann S. Highly Sensitive Feature Detection for High Resolution LC/MS. BMC Bioinformatics 2008, 9, 504 10.1186/1471-2105-9-504. 19040729 PMC2639432

[ref58] Züllig T.; Trötzmüller M.; Köfeler H. C. Lipidomics from Sample Preparation to Data Analysis: A Primer. Anal Bioanal Chem. 2020, 412 (10), 2191–2209. 10.1007/s00216-019-02241-y. 31820027 PMC7118050

[ref59] Du X.; Smirnov A.; Pluskal T.; Jia W.; Sumner S. Metabolomics Data Preprocessing Using ADAP and MZmine 2. In Methods Mol. Biol.; Li S., Ed.; Humana Press Inc.: New York, NY, USA, 2020; Vol. 2104, pp 25–48. 10.1007/978-1-0716-0239-3_3 PMC8359540 31953811

[ref60] Goracci L.; Tortorella S.; Tiberi P.; Pellegrino R. M.; di Veroli A.; Valeri A.; Cruciani G. Lipostar, a Comprehensive Platform-Neutral Cheminformatics Tool for Lipidomics. Anal. Chem. 2017, 89 (11), 6257–6264. 10.1021/acs.analchem.7b01259. 28471643

[ref61] Windig W.; Phalp J. M.; Payne A. W. A Noise and Background Reduction Method for Component Detection in Liquid Chromatography/Mass Spectrometry. Anal. Chem. 1996, 68 (20), 3602–3606. 10.1021/ac960435y.

[ref62] Tsugawa H.; Cajka T.; Kind T.; Ma Y.; Higgins B.; Ikeda K.; Kanazawa M.; Vandergheynst J.; Fiehn O.; Arita M. MS-DIAL: Data-Independent MS/MS Deconvolution for Comprehensive Metabolome Analysis. Nat. Methods 2015, 12 (6), 523–526. 10.1038/nmeth.3393. 25938372 PMC4449330

[ref63] Pluskal T.; Castillo S.; Villar-Briones A.; Orešič M. MZmine 2: Modular Framework for Processing, Visualizing, and Analyzing Mass Spectrometry-Based Molecular Profile Data. BMC Bioinformatics 2010, 11 (1), 395 10.1186/1471-2105-11-395. 20650010 PMC2918584

[ref64] Hartler J.; Trötzmüller M.; Chitraju C.; Spener F.; Köfeler H. C.; Thallinger G. G. Lipid Data Analyzer: Unattended Identification and Quantitation of Lipids in LC-MS Data. Bioinformatics 2011, 27 (4), 572–577. 10.1093/bioinformatics/btq699. 21169379

[ref65] Myers O. D.; Sumner S. J.; Li S.; Barnes S.; Du X. Detailed Investigation and Comparison of the XCMS and MZmine 2 Chromatogram Construction and Chromatographic Peak Detection Methods for Preprocessing Mass Spectrometry Metabolomics Data. Anal. Chem. 2017, 89 (17), 8689–8695. 10.1021/acs.analchem.7b01069. 28752757

[ref66] el Abiead Y.; Milford M.; Schoeny H.; Rusz M.; Salek R. M.; Koellensperger G. Power of MzRAPP-Based Performance Assessments in MS1-Based Nontargeted Feature Detection. Anal. Chem. 2022, 94 (24), 8588–8595. 10.1021/acs.analchem.1c05270. 35671103 PMC9218958

[ref67] de Vijlder T.; Valkenborg D.; Lemière F.; Romijn E. P.; Laukens K.; Cuyckens F. A Tutorial in Small Molecule Identification via Electrospray Ionization-Mass Spectrometry: The Practical Art of Structural Elucidation. Mass Spectrom Rev. 2018, 37 (5), 607–629. 10.1002/mas.21551. 29120505 PMC6099382

[ref68] Kind T.; Fiehn O. Metabolomic Database Annotations via Query of Elemental Compositions: Mass Accuracy Is Insufficient Even at Less than 1 Ppm. BMC Bioinformatics 2006, 7, 234 10.1186/1471-2105-7-234. 16646969 PMC1464138

[ref69] Köfeler H. C.; Eichmann T. O.; Ahrends R.; Bowden J. A.; Danne-Rasche N.; Dennis E. A.; Fedorova M.; Griffiths W. J.; Han X.; Hartler J.; Holčapek M.; Jirásko R.; Koelmel J. P.; Ejsing C. S.; Liebisch G.; Ni Z.; O’Donnell V. B.; Quehenberger O.; Schwudke D.; Shevchenko A.; Wakelam M. J. O.; Wenk M. R.; Wolrab D.; Ekroos K. Quality Control Requirements for the Correct Annotation of Lipidomics Data. Nat. Commun. 2021, 12 (1), 4771 10.1038/s41467-021-24984-y. 34362906 PMC8346590

[ref70] Kind T.; Liu K. H.; Lee D. Y.; Defelice B.; Meissen J. K.; Fiehn O. LipidBlast in Silico Tandem Mass Spectrometry Database for Lipid Identification. Nat. Methods 2013, 10 (8), 755–758. 10.1038/nmeth.2551. 23817071 PMC3731409

[ref71] Hutchins P. D.; Russell J. D.; Coon J. J. Mapping Lipid Fragmentation for Tailored Mass Spectral Libraries. J. Am. Soc. Mass Spectrom. 2019, 30 (4), 659–668. 10.1007/s13361-018-02125-y. 30756325 PMC6447430

[ref72] Chen X.; Yin Y.; Zhou Z.; Li T.; Zhu Z. J. Development of a Combined Strategy for Accurate Lipid Structural Identification and Quantification in Ion-Mobility Mass Spectrometry Based Untargeted Lipidomics. Anal. Chim. Acta 2020, 1136, 115–124. 10.1016/j.aca.2020.08.048. 33081935

[ref73] Korf A.; Jeck V.; Schmid R.; Helmer P. O.; Hayen H. Lipid Species Annotation at Double Bond Position Level with Custom Databases by Extension of the MZmine 2 Open-Source Software Package. Anal. Chem. 2019, 91 (8), 5098–5105. 10.1021/acs.analchem.8b05493. 30892876

[ref74] Cajka T.; Fiehn O. Toward Merging Untargeted and Targeted Methods in Mass Spectrometry-Based Metabolomics and Lipidomics. Anal. Chem. 2016, 88 (1), 524–545. 10.1021/acs.analchem.5b04491. 26637011

[ref75] Balazy M. Eicosanomics: Targeted Lipidomics of Eicosanoids in Biological Systems. Prostaglandins Other Lipid Mediat 2004, 73 (3–4), 173–180. 10.1016/j.prostaglandins.2004.03.003. 15287150

[ref76] Köfeler H. C.; Ahrends R.; Baker E. S.; Ekroos K.; Han X.; Hoffmann N.; Holčapek M.; Wenk M. R.; Liebisch G. Recommendations for Good Practice in MS-Based Lipidomics. J. Lipid Res. 2021, 62, 100138 10.1016/j.jlr.2021.100138. 34662536 PMC8585648

[ref77] Xu H.; Valenzuela N.; Fai S.; Figeys D.; Bennett S. A. L. Targeted Lipidomics - Advances in Profiling Lysophosphocholine and Platelet-Activating Factor Second Messengers. FEBS J. 2013, 280 (22), 5652–5667. 10.1111/febs.12423. 23826908

[ref78] Criscuolo A.; Nepachalovich P.; Fernando D.; Rio G.-D.; Lange M.; Ni Z.; Blüher M.; Fedorova M. Epilipidomics Platform for Holistic Profiling of Oxidized Complex Lipids in Blood Plasma of Obese Individuals. bioRxiv 2021, 2021.12.23.473968 10.1101/2021.12.23.473968.

[ref79] Lerno L. A.; German J. B.; Lebrilla C. B. Method for the Identification of Lipid Classes Based on Referenced Kendrick Mass Analysis. Anal. Chem. 2010, 82 (10), 4236–4245. 10.1021/ac100556g. 20426402 PMC2875411

[ref80] Sleno L. The Use of Mass Defect in Modern Mass Spectrometry. J. Mass Spectrom 2012, 47 (2), 226–236. 10.1002/jms.2953. 22359333

[ref81] Fouquet T. N. J. The Kendrick Analysis for Polymer Mass Spectrometry. J. Mass Spectrom 2019, 54 (12), 933–947. 10.1002/jms.4480. 31758605

[ref82] Korf A.; Vosse C.; Schmid R.; Helmer P. O.; Jeck V.; Hayen H. Three-Dimensional Kendrick Mass Plots as a Tool for Graphical Lipid Identification. Rapid Commun. Mass Spectrom. 2018, 32 (12), 981–991. 10.1002/rcm.8117. 29575335

[ref83] Helmer P. O.; Korf A.; Hayen H. Analysis of Artificially Oxidized Cardiolipins and Monolyso-Cardiolipins via Liquid Chromatography/High-Resolution Mass Spectrometry and Kendrick Mass Defect Plots after Hydrophilic Interaction Liquid Chromatography Based Sample Preparation. Rapid Commun. Mass Spectrom. 2020, 34 (1), e8566 10.1002/rcm.8566. 31469924

[ref84] Müller W. H.; Verdin A.; Kune C.; Far J.; de Pauw E.; Malherbe C.; Eppe G. Dual-Polarity SALDI FT-ICR MS Imaging and Kendrick Mass Defect Data Filtering for Lipid Analysis. Anal Bioanal Chem. 2021, 413 (10), 2821–2830. 10.1007/s00216-020-03020-w. 33125540

[ref85] Korf A.; Fouquet T.; Schmid R.; Hayen H.; Hagenhoff S. Expanding the Kendrick Mass Plot Toolbox in MZmine 2 to Enable Rapid Polymer Characterization in Liquid Chromatography-Mass Spectrometry Data Sets. Anal. Chem. 2020, 92 (1), 628–633. 10.1021/acs.analchem.9b03863. 31801022

[ref86] Krzywinski M.; Schein J.; Birol I.; Connors J.; Gascoyne R.; Horsman D.; Jones S. J.; Marra M. A. Circos: An Information Aesthetic for Comparative Genomics. Genome Res. 2009, 19 (9), 1639–1645. 10.1101/gr.092759.109. 19541911 PMC2752132

[ref87] Jha P.; McDevitt M. T.; Gupta R.; Quiros P. M.; Williams E. G.; Gariani K.; Sleiman M. B.; Diserens L.; Jochem A.; Ulbrich A.; Coon J. J.; Auwerx J.; Pagliarini D. J. Systems Analyses Reveal Physiological Roles and Genetic Regulators of Liver Lipid Species. Cell Syst 2018, 6 (6), 722–733.e6. 10.1016/j.cels.2018.05.016. 29909277 PMC6054463

[ref88] Zhang H.; Meltzer P.; Davis S. RCircos: An R Package for Circos 2D Track Plots. BMC Bioinformatics 2013, 14 (1), 244 10.1186/1471-2105-14-244. 23937229 PMC3765848

[ref89] Cui Y.; Chen X.; Luo H.; Fan Z.; Luo J.; He S.; Yue H.; Zhang P.; Chen R. BioCircos.Js: An Interactive Circos JavaScript Library for Biological Data Visualization on Web Applications. Bioinformatics 2016, 32 (11), 1740–1742. 10.1093/bioinformatics/btw041. 26819473

[ref90] Collins J. R.; Edwards B. R.; Fredricks H. F.; van Mooy B. A. S. LOBSTAHS: An Adduct-Based Lipidomics Strategy for Discovery and Identification of Oxidative Stress Biomarkers. Anal. Chem. 2016, 88 (14), 7154–7162. 10.1021/acs.analchem.6b01260. 27322848

[ref91] Koelmel J. P.; Kroeger N. M.; Ulmer C. Z.; Bowden J. A.; Patterson R. E.; Cochran J. A.; Beecher C. W. W.; Garrett T. J.; Yost R. A. LipidMatch: An Automated Workflow for Rule-Based Lipid Identification Using Untargeted High-Resolution Tandem Mass Spectrometry Data. BMC Bioinformatics 2017, 18 (1), 331 10.1186/s12859-017-1744-3. 28693421 PMC5504796

[ref92] Mohamed A.; Molendijk J.; Hill M. M. Lipidr: A Software Tool for Data Mining and Analysis of Lipidomics Datasets. J. Proteome Res. 2020, 19 (7), 2890–2897. 10.1021/acs.jproteome.0c00082. 32168452

[ref93] O’Connor A.; Brasher C. J.; Slatter D. A.; Meckelmann S. W.; Hawksworth J. I.; Allen S. M.; O’Donnell V. B. LipidFinder: A Computational Workflow for Discovery of Lipids Identifies Eicosanoid-Phosphoinositides in Platelets. JCI Insight 2017, 2 (7), e91634 10.1172/jci.insight.91634. 28405621 PMC5374061

[ref94] Ross D. H.; Cho J. H.; Zhang R.; Hines K. M.; Xu L. LiPydomics: A Python Package for Comprehensive Prediction of Lipid Collision Cross Sections and Retention Times and Analysis of Ion Mobility-Mass Spectrometry-Based Lipidomics Data. Anal. Chem. 2020, 92 (22), 14967–14975. 10.1021/acs.analchem.0c02560. 33119270 PMC7816765

[ref95] Türei D. lipyd: a Python module for lipidomics LC MS/MS data analysis. https://saezlab.github.io/lipyd/ (accessed 2022-10-09).

[ref96] Krettler C. A.; Hartler J.; Thallinger G. G. Identification and Quantification of Oxidized Lipids in LC-MS Lipidomics Data. Stud Health Technol. Inform 2020, 271, 39–48. 10.3233/SHTI200072. 32578539

[ref97] Koelmel J. P. LipidMatch Flow - Innovative Omics. https://innovativeomics.com/software/lipidmatch-flow-covers-entire-lipidomics-workflow/ (accessed 2022-10-09).

[ref98] Tsugawa H.; Ikeda K.; Takahashi M.; Satoh A.; Mori Y.; Uchino H.; Okahashi N.; Yamada Y.; Tada I.; Bonini P.; Higashi Y.; Okazaki Y.; Zhou Z.; Zhu Z. J.; Koelmel J.; Cajka T.; Fiehn O.; Saito K.; Arita M.; Arita M. A Lipidome Atlas in MS-DIAL 4. Nat. Biotechnol. 2020, 38 (10), 1159–1163. 10.1038/s41587-020-0531-2. 32541957

[ref99] Pluskal T.; Korf A.; Smirnov A.; Schmid R.; Fallon T. R.; Du X.; Weng J. K. CHAPTER 7: Metabolomics Data Analysis Using MZmine. In New Developments in Mass Spectrometry; Royal Society of Chemistry, 2020; pp 232–254 10.1039/9781788019880-00232.

[ref100] Wei R.; Wang J.; Su M.; Jia E.; Chen S.; Chen T.; Ni Y. Missing Value Imputation Approach for Mass Spectrometry-Based Metabolomics Data. Sci. Rep 2018, 8 (1), 663 10.1038/s41598-017-19120-0. 29330539 PMC5766532

[ref101] Guijas C.; Montenegro-Burke J. R.; Domingo-Almenara X.; Palermo A.; Warth B.; Hermann G.; Koellensperger G.; Huan T.; Uritboonthai W.; Aisporna A. E.; Wolan D. W.; Spilker M. E.; Benton H. P.; Siuzdak G. METLIN: A Technology Platform for Identifying Knowns and Unknowns. Anal. Chem. 2018, 90 (5), 3156–3164. 10.1021/acs.analchem.7b04424. 29381867 PMC5933435

[ref102] Horai H.; Arita M.; Kanaya S.; Nihei Y.; Ikeda T.; Suwa K.; Ojima Y.; Tanaka K.; Tanaka S.; Aoshima K.; Oda Y.; Kakazu Y.; Kusano M.; Tohge T.; Matsuda F.; Sawada Y.; Hirai M. Y.; Nakanishi H.; Ikeda K.; Akimoto N.; Maoka T.; Takahashi H.; Ara T.; Sakurai N.; Suzuki H.; Shibata D.; Neumann S.; Iida T.; Tanaka K.; Funatsu K.; Matsuura F.; Soga T.; Taguchi R.; Saito K.; Nishioka T. MassBank: A Public Repository for Sharing Mass Spectral Data for Life Sciences. J. Mass Spectrom 2010, 45 (7), 703–714. 10.1002/jms.1777. 20623627

[ref103] MassBank of North America. https://mona.fiehnlab.ucdavis.edu/ (accessed 2022-10-09).

[ref104] Degtyarenko K.; de Matos P.; Ennis M.; Hastings J.; Zbinden M.; McNaught A.; Alcantara R.; Darsow M.; Guedj M.; Ashburner M. ChEBI: A Database and Ontology for Chemical Entities of Biological Interest. Nucleic Acids Res. 2007, 36, D344–D350. 10.1093/nar/gkm791. 17932057 PMC2238832

[ref105] Kim S.; Chen J.; Cheng T.; Gindulyte A.; He J.; He S.; Li Q.; Shoemaker B. A.; Thiessen P. A.; Yu B.; Zaslavsky L.; Zhang J.; Bolton E. E. PubChem in 2021: New Data Content and Improved Web Interfaces. Nucleic Acids Res. 2021, 49 (D1), D1388–D1395. 10.1093/nar/gkaa971. 33151290 PMC7778930

[ref106] Fahy E.; Sud M.; Cotter D.; Subramaniam S. LIPID MAPS Online Tools for Lipid Research. Nucleic Acids Res. 2007, 35, W606–W612. 10.1093/nar/gkm324. 17584797 PMC1933166

[ref107] Aimo L.; Liechti R.; Hyka-Nouspikel N.; Niknejad A.; Gleizes A.; Götz L.; Kuznetsov D.; David F. P. A.; van der Goot F. G.; Riezman H.; Bougueleret L.; Xenarios I.; Bridge A. The SwissLipids Knowledgebase for Lipid Biology. Bioinformatics 2015, 31 (17), 2860–2866. 10.1093/bioinformatics/btv285. 25943471 PMC4547616

[ref108] O’Donnell V. B.; Dennis E. A.; Wakelam M. J. O.; Subramaniam S. LIPID MAPS: Serving the next Generation of Lipid Researchers with Tools, Resources, Data, and Training. Sci. Signal 2019, 12 (563), eaaw2964 10.1126/scisignal.aaw2964. 30622195

[ref109] Wishart D. S.; Guo A. C.; Oler E.; Wang F.; Anjum A.; Peters H.; Dizon R.; Sayeeda Z.; Tian S.; Lee B. L.; Berjanskii M.; Mah R.; Yamamoto M.; Jovel J.; Torres-Calzada C.; Hiebert-Giesbrecht M.; Lui V. W.; Varshavi D.; Varshavi D.; Allen D.; Arndt D.; Khetarpal N.; Sivakumaran A.; Harford K.; Sanford S.; Yee K.; Cao X.; Budinski Z.; Liigand J.; Zhang L.; Zheng J.; Mandal R.; Karu N.; Dambrova M.; Schiöth H. B.; Greiner R.; Gautam V. HMDB 5.0: The Human Metabolome Database for 2022. Nucleic Acids Res. 2022, 50 (D1), D622–D631. 10.1093/nar/gkab1062. 34986597 PMC8728138

[ref110] Kuo T. C.; Tseng Y. J. LipidPedia: A Comprehensive Lipid Knowledgebase. Bioinformatics 2018, 34 (17), 2982–2987. 10.1093/bioinformatics/bty213. 29648583 PMC6129305

[ref111] Watanabe K.; Yasugi E.; Oshima M. How to Search the Glycolipid Data in “LIPID BANK for Web”, the Newly Developed Lipid Database in Japan. Trends Glycosci Glycotechnol 2000, 12 (65), 175–184. 10.4052/tigg.12.175.

[ref112] Gil-De-La-Fuente A.; Godzien J.; Saugar S.; Garcia-Carmona R.; Badran H.; Wishart D. S.; Barbas C.; Otero A. CEU Mass Mediator 3.0: A Metabolite Annotation Tool. J. Proteome Res. 2019, 18 (2), 797–802. 10.1021/acs.jproteome.8b00720. 30574788

[ref113] Lauder S. N.; Allen-Redpath K.; Slatter D. A.; Aldrovandi M.; O’Connor A.; Farewell D.; Percy C. L.; Molhoek J. E.; Rannikko S.; Tyrrell V. J.; Ferla S.; Milne G. L.; Poole A. W.; Thomas C. P.; Obaji S.; Taylor P. R.; Jones S. A.; de Groot P. G.; Urbanus R. T.; Hörkkö S.; Uderhardt S.; Ackermann J.; Vince Jenkins P.; Brancale A.; Krönke G.; Collins P. W.; O’Donnell V. B. Networks of Enzymatically Oxidized Membrane Lipids Support Calcium-Dependent Coagulation Factor Binding to Maintain Hemostasis. Sci. Signal 2017, 10 (507), eaan2787 10.1126/scisignal.aan2787. 29184033 PMC5720345

[ref114] Köhler N.; Rose T. D.; Falk L.; Pauling J. K. Investigating Global Lipidome Alterations with the Lipid Network Explorer. Metabolites 2021, 11 (8), 488 10.3390/metabo11080488. 34436429 PMC8398636

[ref115] Rose T. D.; Köhler N.; Falk L.; Klischat L.; Lazareva O. E.; Pauling J. K. Lipid Network and Moiety Analysis for Revealing Enzymatic Dysregulation and Mechanistic Alterations from Lipidomics Data. bioRxiv 2022, 2022.02.04.479101 10.1101/2022.02.04.479101. PMC9851308 36592059

[ref116] Bansal P.; Morgat A.; Axelsen K. B.; Muthukrishnan V.; Coudert E.; Aimo L.; Hyka-Nouspikel N.; Gasteiger E.; Kerhornou A.; Neto T. B.; Pozzato M.; Blatter M. C.; Ignatchenko A.; Redaschi N.; Bridge A. Rhea, the Reaction Knowledgebase in 2022. Nucleic Acids Res. 2022, 50 (D1), D693–D700. 10.1093/nar/gkab1016. 34755880 PMC8728268

[ref117] Gillespie M.; Jassal B.; Stephan R.; Milacic M.; Rothfels K.; Senff-Ribeiro A.; Griss J.; Sevilla C.; Matthews L.; Gong C.; Deng C.; Varusai T.; Ragueneau E.; Haider Y.; May B.; Shamovsky V.; Weiser J.; Brunson T.; Sanati N.; Beckman L.; Shao X.; Fabregat A.; Sidiropoulos K.; Murillo J.; Viteri G.; Cook J.; Shorser S.; Bader G.; Demir E.; Sander C.; Haw R.; Wu G.; Stein L.; Hermjakob H.; D’Eustachio P. The Reactome Pathway Knowledgebase 2022. Nucleic Acids Res. 2022, 50 (D1), D687–D692. 10.1093/nar/gkab1028. 34788843 PMC8689983

[ref118] Aron A. T.; Gentry E. C.; McPhail K. L.; Nothias L. F.; Nothias-Esposito M.; Bouslimani A.; Petras D.; Gauglitz J. M.; Sikora N.; Vargas F.; van der Hooft J. J. J.; Ernst M.; Kang K. bin; Aceves C. M.; Caraballo-Rodríguez A. M.; Koester I.; Weldon K. C.; Bertrand S.; Roullier C.; Sun K.; Tehan R. M.; Boya P C. A.; Christian M. H.; Gutiérrez M.; Ulloa A. M.; Tejeda Mora J. A.; Mojica-Flores R.; Lakey-Beitia J.; Vásquez-Chaves V.; Zhang Y.; Calderón A. I.; Tayler N.; Keyzers R. A.; Tugizimana F.; Ndlovu N.; Aksenov A. A.; Jarmusch A. K.; Schmid R.; Truman A. W.; Bandeira N.; Wang M.; Dorrestein P. C. Reproducible Molecular Networking of Untargeted Mass Spectrometry Data Using GNPS. Nat. Protoc 2020, 15 (6), 1954–1991. 10.1038/s41596-020-0317-5. 32405051

[ref119] Nothias L. F.; Petras D.; Schmid R.; Dührkop K.; Rainer J.; Sarvepalli A.; Protsyuk I.; Ernst M.; Tsugawa H.; Fleischauer M.; Aicheler F.; Aksenov A. A.; Alka O.; Allard P. M.; Barsch A.; Cachet X.; Caraballo-Rodriguez A. M.; da Silva R. R.; Dang T.; Garg N.; Gauglitz J. M.; Gurevich A.; Isaac G.; Jarmusch A. K.; Kameník Z.; Kang K. bin; Kessler N.; Koester I.; Korf A.; le Gouellec A.; Ludwig M.; Martin H C.; McCall L. I.; McSayles J.; Meyer S. W.; Mohimani H.; Morsy M.; Moyne O.; Neumann S.; Neuweger H.; Nguyen N. H.; Nothias-Esposito M.; Paolini J.; Phelan V. v.; Pluskal T.; Quinn R. A.; Rogers S.; Shrestha B.; Tripathi A.; van der Hooft J. J. J.; Vargas F.; Weldon K. C.; Witting M.; Yang H.; Zhang Z.; Zubeil F.; Kohlbacher O.; Böcker S.; Alexandrov T.; Bandeira N.; Wang M.; Dorrestein P. C. Feature-Based Molecular Networking in the GNPS Analysis Environment. Nat. Methods 2020, 17 (9), 905–908. 10.1038/s41592-020-0933-6. 32839597 PMC7885687

[ref120] Wang M.; Carver J. J.; Phelan V. v.; Sanchez L. M.; Garg N.; Peng Y.; Nguyen D. D.; Watrous J.; Kapono C. A.; Luzzatto-Knaan T.; Porto C.; Bouslimani A.; Melnik A. v.; Meehan M. J.; Liu W. T.; Crüsemann M.; Boudreau P. D.; Esquenazi E.; Sandoval-Calderón M.; Kersten R. D.; Pace L. A.; Quinn R. A.; Duncan K. R.; Hsu C. C.; Floros D. J.; Gavilan R. G.; Kleigrewe K.; Northen T.; Dutton R. J.; Parrot D.; Carlson E. E.; Aigle B.; Michelsen C. F.; Jelsbak L.; Sohlenkamp C.; Pevzner P.; Edlund A.; McLean J.; Piel J.; Murphy B. T.; Gerwick L.; Liaw C. C.; Yang Y. L.; Humpf H. U.; Maansson M.; Keyzers R. A.; Sims A. C.; Johnson A. R.; Sidebottom A. M.; Sedio B. E.; Klitgaard A.; Larson C. B.; Boya C. A. P.; Torres-Mendoza D.; Gonzalez D. J.; Silva D. B.; Marques L. M.; Demarque D. P.; Pociute E.; O’Neill E. C.; Briand E.; Helfrich E. J. N.; Granatosky E. A.; Glukhov E.; Ryffel F.; Houson H.; Mohimani H.; Kharbush J. J.; Zeng Y.; Vorholt J. A.; Kurita K. L.; Charusanti P.; McPhail K. L.; Nielsen K. F.; Vuong L.; Elfeki M.; Traxler M. F.; Engene N.; Koyama N.; Vining O. B.; Baric R.; Silva R. R.; Mascuch S. J.; Tomasi S.; Jenkins S.; Macherla V.; Hoffman T.; Agarwal V.; Williams P. G.; Dai J.; Neupane R.; Gurr J.; Rodríguez A. M. C.; Lamsa A.; Zhang C.; Dorrestein K.; Duggan B. M.; Almaliti J.; Allard P. M.; Phapale P.; Nothias L. F.; Alexandrov T.; Litaudon M.; Wolfender J. L.; Kyle J. E.; Metz T. O.; Peryea T.; Nguyen D. T.; VanLeer D.; Shinn P.; Jadhav A.; Müller R.; Waters K. M.; Shi W.; Liu X.; Zhang L.; Knight R.; Jensen P. R.; Palsson B.; Pogliano K.; Linington R. G.; Gutiérrez M.; Lopes N. P.; Gerwick W. H.; Moore B. S.; Dorrestein P. C.; Bandeira N. Sharing and Community Curation of Mass Spectrometry Data with Global Natural Products Social Molecular Networking. Nat. Biotechnol. 2016, 34 (8), 828–837. 10.1038/nbt.3597. 27504778 PMC5321674

[ref121] Watrous J. D.; Niiranen T. J.; Lagerborg K. A.; Henglin M.; Xu Y. J.; Rong J.; Sharma S.; Vasan R. S.; Larson M. G.; Armando A.; Mora S.; Quehenberger O.; Dennis E. A.; Cheng S.; Jain M. Directed Non-Targeted Mass Spectrometry and Chemical Networking for Discovery of Eicosanoids and Related Oxylipins. Cell Chem. Biol. 2019, 26 (3), 433–442.e4. 10.1016/j.chembiol.2018.11.015. 30661990 PMC6636917

[ref122] Magny R.; Regazzetti A.; Kessal K.; Genta-Jouve G.; Baudouin C.; Mélik-Parsadaniantz S.; Brignole-Baudouin F.; Laprévote O.; Auzeil N. Lipid Annotation by Combination of UHPLC-HRMS (MS), Molecular Networking, and Retention Time Prediction: Application to a Lipidomic Study of In Vitro Models of Dry Eye Disease. Metabolites 2020, 10 (6), 225 10.3390/metabo10060225. 32486009 PMC7345884

[ref123] Okahashi N.; Ueda M.; Yasuda S.; Tsugawa H.; Arita M. Global Profiling of Gut Microbiota-Associated Lipid Metabolites in Antibiotic-Treated Mice by LC-MS/MS-Based Analyses. STAR Protoc 2021, 2 (2), 100492 10.1016/j.xpro.2021.100492. 33997812 PMC8091925

[ref124] Ding S.; Bale N. J.; Hopmans E. C.; Villanueva L.; Arts M. G. I.; Schouten S.; Sinninghe Damsté J. S. Lipidomics of Environmental Microbial Communities. II: Characterization Using Molecular Networking and Information Theory. Front Microbiol 2021, 12, 659315 10.3389/fmicb.2021.659315. 34322097 PMC8311935

[ref125] Chen X.; Peng X.; Sun X.; Pan L.; Shi J.; Gao Y.; Lei Y.; Jiang F.; Li R.; Liu Y.; Xu Y. J. Development and Application of Feature-Based Molecular Networking for Phospholipidomics Analysis. J. Agric. Food Chem. 2022, 70 (25), 7815–7825. 10.1021/acs.jafc.2c01770. 35709392

[ref126] della Sala G.; Coppola D.; Virgili R.; Vitale G. A.; Tanduo V.; Teta R.; Crocetta F.; de Pascale D. Untargeted Metabolomics Yields Insights Into the Lipidome of Botrylloides Niger Herdman, 1886, An Ascidian Invading the Mediterranean Sea. Front Mar Sci. 2022, 9, 865751 10.3389/fmars.2022.865751.

[ref127] Olivon F.; Elie N.; Grelier G.; Roussi F.; Litaudon M.; Touboul D. MetGem Software for the Generation of Molecular Networks Based on the T-SNE Algorithm. Anal. Chem. 2018, 90 (23), 13900–13908. 10.1021/acs.analchem.8b03099. 30335965

[ref128] Woldegebriel M.; Derks E. Artificial Neural Network for Probabilistic Feature Recognition in Liquid Chromatography Coupled to High-Resolution Mass Spectrometry. Anal. Chem. 2017, 89 (2), 1212–1221. 10.1021/acs.analchem.6b03678. 28035799

[ref129] Melnikov A. D.; Tsentalovich Y. P.; Yanshole V. v. Deep Learning for the Precise Peak Detection in High-Resolution LC-MS Data. Anal. Chem. 2020, 92 (1), 588–592. 10.1021/acs.analchem.9b04811. 31841624

[ref130] Guo J.; Shen S.; Xing S.; Chen Y.; Chen F.; Porter E. M.; Yu H.; Huan T. EVA: Evaluation of Metabolic Feature Fidelity Using a Deep Learning Model Trained With Over 25000 Extracted Ion Chromatograms. Anal. Chem. 2021, 93 (36), 12181–12186. 10.1021/acs.analchem.1c01309. 34455775

[ref131] Bueschl C.; Doppler M.; Varga E.; Seidl B.; Flasch M.; Warth B.; Zanghellini J. PeakBot: Machine Learning Based Chromatographic Peak Picking. Bioinformatics 2022, 38 (13), 3422–3428. 10.1093/bioinformatics/btac344. 35604083 PMC9237678

[ref132] Witting M.; Böcker S. Current Status of Retention Time Prediction in Metabolite Identification. J. Sep Sci. 2020, 43 (9–10), 1746–1754. 10.1002/jssc.202000060. 32144942

[ref133] Zhou Z.; Tu J.; Zhu Z. J. Advancing the Large-Scale CCS Database for Metabolomics and Lipidomics at the Machine-Learning Era. Curr. Opin Chem. Biol. 2018, 42, 34–41. 10.1016/j.cbpa.2017.10.033. 29136580

[ref134] Zhou Z.; Tu J.; Xiong X.; Shen X.; Zhu Z. J. LipidCCS: Prediction of Collision Cross-Section Values for Lipids with High Precision To Support Ion Mobility-Mass Spectrometry-Based Lipidomics. Anal. Chem. 2017, 89 (17), 9559–9566. 10.1021/acs.analchem.7b02625. 28764323

[ref135] Dührkop K.; Fleischauer M.; Ludwig M.; Aksenov A. A.; Melnik A. v.; Meusel M.; Dorrestein P. C.; Rousu J.; Böcker S. SIRIUS 4: A Rapid Tool for Turning Tandem Mass Spectra into Metabolite Structure Information. Nat. Methods 2019, 16 (4), 299–302. 10.1038/s41592-019-0344-8. 30886413

[ref136] Stravs M. A.; Dührkop K.; Böcker S.; Zamboni N. MSNovelist: De Novo Structure Generation from Mass Spectra. Nat. Methods 2022, 19 (7), 865–870. 10.1038/s41592-022-01486-3. 35637304 PMC9262714

[ref137] boecker-lab/sirius: SIRIUS is a software for discovering a landscape of de-novo identification of metabolites using tandem mass spectrometry. https://github.com/boecker-Lab/sirius (accessed 2022-10-09).

[ref138] Wang M.; Jarmusch A. K.; Vargas F.; Aksenov A. A.; Gauglitz J. M.; Weldon K.; Petras D.; da Silva R.; Quinn R.; Melnik A. v.; van der Hooft J. J. J.; Caraballo-Rodríguez A. M.; Nothias L. F.; Aceves C. M.; Panitchpakdi M.; Brown E.; di Ottavio F.; Sikora N.; Elijah E. O.; Labarta-Bajo L.; Gentry E. C.; Shalapour S.; Kyle K. E.; Puckett S. P.; Watrous J. D.; Carpenter C. S.; Bouslimani A.; Ernst M.; Swafford A. D.; Zúñiga E. I.; Balunas M. J.; Klassen J. L.; Loomba R.; Knight R.; Bandeira N.; Dorrestein P. C. Mass Spectrometry Searches Using MASST. Nat. Biotechnol. 2020, 38 (1), 23–26. 10.1038/s41587-019-0375-9. 31894142 PMC7236533

[ref139] Jarmusch A. K.; Aron A. T.; Petras D.; Phelan V. v.; Bittremieux W.; Acharya D. D.; Ahmed M. M. A.; Bauermeister A.; Bertin M. J.; Boudreau P. D.; Borges R. M.; Bowen B. P.; Brown C. J.; Chagas F. O.; Clevenger K. D.; Correia M. S. P.; Crandall W. J.; Crüsemann M.; Damiani T.; Fiehn O.; Garg N.; Gerwick W. H.; Gilbert J. R.; Globisch D.; Gomes P. W. P.; Heuckeroth S.; James C. A.; Jarmusch S. A.; Kakhkhorov S. A.; Kang K. bin.; Kersten R. D.; Kim H.; Kirk R. D.; Kohlbacher O.; Kontou E. E.; Liu K.; Lizama-Chamu I.; Luu G. T.; Knaan T. L.; Marty M. T.; McAvoy A. C.; McCall L.-I.; Mohamed O. G.; Nahor O.; Niedermeyer T. H. J.; Northen T. R.; Overdahl K. E.; Pluskal T.; Rainer J.; Reher R.; Rodriguez E.; Sachsenberg T. T.; Sanchez L. M.; Schmid R.; Stevens C.; Tian Z.; Tripathi A.; Tsugawa H.; Nishida K.; Matsuzawa Y.; Hooft J. J. J. van der.; Vicini A.; Walter A.; Weber T.; Xiong Q.; Xu T.; Zhao H. N.; Dorrestein P. C.; Wang M. A Universal Language for Finding Mass Spectrometry Data Patterns. bioRxiv 2022, 2022.08.06.503000 10.1101/2022.08.06.503000.

[ref140] GNPS MASST. https://masst.ucsd.edu/ (accessed 2022-10-09).

[ref141] Sud M.; Fahy E.; Cotter D.; Azam K.; Vadivelu I.; Burant C.; Edison A.; Fiehn O.; Higashi R.; Nair K. S.; Sumner S.; Subramaniam S. Metabolomics Workbench: An International Repository for Metabolomics Data and Metadata, Metabolite Standards, Protocols, Tutorials and Training, and Analysis Tools. Nucleic Acids Res. 2016, 44 (D1), D463–D470. 10.1093/nar/gkv1042. 26467476 PMC4702780

[ref142] Haug K.; Salek R. M.; Conesa P.; Hastings J.; de Matos P.; Rijnbeek M.; Mahendraker T.; Williams M.; Neumann S.; Rocca-Serra P.; Maguire E.; González-Beltrán A.; Sansone S.-A.; Griffin J. L.; Steinbeck C. MetaboLights—an Open-Access General-Purpose Repository for Metabolomics Studies and Associated Meta-Data. Nucleic Acids Res. 2013, 41 (D1), D781–D786. 10.1093/nar/gks1004. 23109552 PMC3531110

[ref143] Godzien J.; Ciborowski M.; Martínez-Alcázar M. P.; Samczuk P.; Kretowski A.; Barbas C. Rapid and Reliable Identification of Phospholipids for Untargeted Metabolomics with LC-ESI-QTOF-MS/MS. J. Proteome Res. 2015, 14 (8), 3204–3216. 10.1021/acs.jproteome.5b00169. 26080858

[ref144] Zhou Z.; Luo M.; Chen X.; Yin Y.; Xiong X.; Wang R.; Zhu Z.-J. Ion Mobility Collision Cross-Section Atlas for Known and Unknown Metabolite Annotation in Untargeted Metabolomics. Nat. Commun. 2020, 11 (1), 4334 10.1038/s41467-020-18171-8. 32859911 PMC7455731

[ref145] Bonini P.; Kind T.; Tsugawa H.; Barupal D. K.; Fiehn O. Retip: Retention Time Prediction for Compound Annotation in Untargeted Metabolomics. Anal. Chem. 2020, 92 (11), 7515–7522. 10.1021/acs.analchem.9b05765. 32390414 PMC8715951

[ref146] Bach E.; Szedmak S.; Brouard C.; Böcker S.; Rousu J. Liquid-Chromatography Retention Order Prediction for Metabolite Identification. Bioinformatics 2018, 34 (17), i875–i883. 10.1093/bioinformatics/bty590. 30423079

[ref147] Liebal U. W.; Phan A. N. T.; Sudhakar M.; Raman K.; Blank L. M. Machine Learning Applications for Mass Spectrometry-Based Metabolomics. Metabolites 2020, 10 (6), 243 10.3390/metabo10060243. 32545768 PMC7345470

[ref148] Gaud C. C.; Sousa B.; Nguyen A.; Fedorova M.; Ni Z.; O’Donnell V. B.; Wakelam M. J. O.; Andrews S.; Lopez-Clavijo A. F. BioPAN: A Web-Based Tool to Explore Mammalian Lipidome Metabolic Pathways on LIPID MAPS. F1000Res. 2021, 10, 4 10.12688/f1000research.28022.2. 33564392 PMC7848852

