# Correction to “Software and Computational Tools for LC-MS-Based Epilipidomics: Challenges and Solutions”

**DOI:** 10.1021/acs.analchem.3c00305

**Published:** 2023-02-02

**Authors:** Tito Damiani, Stefano Bonciarelli, Gerhard G. Thallinger, Nikolai Koehler, Christoph A. Krettler, Arif K. Salihoğlu, Ansgar Korf, Josch K. Pauling, Tomáš Pluskal, Zhixu Ni, Laura Goracci

**Affiliations:** 1 Institute of Organic Chemistry and Biochemistry of the Czech Academy of Sciences, Flemingovo nám. 2, 160 00 Praha 6, Czech Republic; 2 Department of Chemistry, Biology and Biotechnology, University of Perugia, Via Elce di Sotto 8, 06123 Perugia, Italy; 3 LipiTUM, Chair of Experimental Bioinformatics, Technical University of Munich, Maximus-von-Imhof Forum 3, 85354 Freising, Germany; 4 Enveda Biosciences, Boulder, Colorado 80301, United States; 5 Department of Physiology, Faculty of Medicine and Institute of Health Sciences, Karadeniz Technical University, 61080 Trabzon, Turkey; 6 Bruker Daltonics GmbH & Co. KG, Fahrenheitstraße 4, 28359 Bremen, Germany; 9 Center of Membrane Biochemistry and Lipid Research, Faculty of Medicine Carl Gustav Carus of TU Dresden, 01307 Dresden, Germany; 10 Institute of Biomedical Informatics, Graz University of Technology, 8010 Graz, Austria

The Author Information of the Corresponding Author Zhixu Ni in the original manuscript should be corrected to the following:

Center of Membrane Biochemistry and Lipid Research, Faculty of Medicine Carl Gustav Carus of TU Dresden, 01307 Dresden, Germany

This is corrected in the affiliations of this Correction.

